# Intrapulmonary administration of purified NEIL2 abrogates NF-κB–mediated inflammation

**DOI:** 10.1016/j.jbc.2021.100723

**Published:** 2021-04-28

**Authors:** Nisha Tapryal, Shandy Shahabi, Anirban Chakraborty, Koa Hosoki, Maki Wakamiya, Gobinda Sarkar, Gulshan Sharma, Victor J. Cardenas, Istvan Boldogh, Sanjiv Sur, Gourisankar Ghosh, Tapas K. Hazra

**Affiliations:** 1 Department of Internal Medicine, University of Texas Medical Branch, Galveston, Texas, USA; 2 Department of Chemistry and Biochemistry, University of California, San Diego, La Jolla, California, USA; 3 Department of Medicine, Immunology, Allergy and Rheumatology, Baylor College of Medicine, Houston, Texas, USA; 4 Departments of Biochemistry and Molecular Biology, University of Texas Medical Branch, Galveston, Texas, USA; 5 Department of Orthopedics, Mayo Clinic and Foundation, Rochester, Minnesota, USA; 6 Department of Experimental Pathology, Mayo Clinic and Foundation, Rochester, Minnesota, USA; 7 Department of Microbiology and Immunology, University of Texas Medical Branch, Galveston, Texas, USA

**Keywords:** gene expression, immunosuppression, inflammation, NEIL2, NF-kappa B (NF-κB), BALF, bronchoalveolar lavage fluid, ChIP-qPCR, chromatin immunoprecipitation-qPCR, EMSA, electrophoretic mobility-shift assay, NE, nuclear extract, NEIL2, Nei-like DNA glycosylase 2, NF-κB, nuclear factor kappa B, PBS, phosphate buffered saline, RHR, Rel homology region, RT-qPCR, real-time qPCR, TNFα, tumor necrosis factor α

## Abstract

Aberrant or constitutive activation of nuclear factor kappa B (NF-κB) contributes to various human inflammatory diseases and malignancies *via* the upregulation of genes involved in cell proliferation, survival, angiogenesis, inflammation, and metastasis. Thus, inhibition of NF-κB signaling has potential for therapeutic applications in cancer and inflammatory diseases. We reported previously that Nei-like DNA glycosylase 2 (NEIL2), a mammalian DNA glycosylase, is involved in the preferential repair of oxidized DNA bases from the transcriptionally active sequences *via* the transcription-coupled base excision repair pathway. We have further shown that *Neil2*-null mice are highly sensitive to tumor necrosis factor α (TNFα)- and lipopolysaccharide-induced inflammation. Both TNFα and lipopolysaccharide are potent activators of NF-κB. However, the underlying mechanism of NEIL2's role in the NF-κB–mediated inflammation remains elusive. Here, we have documented a noncanonical function of NEIL2 and demonstrated that the expression of genes, such as *Cxcl1, Cxcl2, Cxcl10, Il6*, and *Tnfα*, involved in inflammation and immune cell migration was significantly higher in both mock- and TNFα-treated *Neil2*-null mice compared with that in the WT mice. NEIL2 blocks NF-κB's binding to target gene promoters by directly interacting with the Rel homology region of RelA and represses proinflammatory gene expression as determined by co-immunoprecipitation, chromatin immunoprecipitation, and electrophoretic mobility-shift assays. Remarkably, intrapulmonary administration of purified NEIL2 *via* a noninvasive nasal route significantly abrogated binding of NF-κB to cognate DNA, leading to decreased expression of proinflammatory genes and neutrophil recruitment in *Neil2*-null as well as WT mouse lungs. Our findings thus highlight the potential of NEIL2 as a biologic for inflammation-associated human diseases.

Oxidative stress and inflammation are normal physiological processes which are balanced in a healthy organism (1). However, under chronic stress, the balance is shifted toward proinflammatory signaling which activates inflammation-inducible genes with subsequent generation of reactive oxygen and nitrogen species (2, 3). These reactive oxygen and nitrogen species can lead to the generation of potentially mutagenic oxidized DNA bases and strand breaks in mammalian genome (4, 5, 6). Such DNA lesions are primarily repaired *via* the base excision repair pathway (7, 8, 9, 10). In recent years, several studies have suggested that the oxidative DNA damage–induced cellular DNA damage response can initiate an immune response that directly activates a variety of transcription factors, such as nuclear factor kappa B (NF-κB), STAT, and interferon regulatory factors (11, 12, 13, 14, 15). Additionally, several groups, including ours, have reported noncanonical roles of the base excision repair proteins, including poly [ADP-ribose] polymerase 1, 8-Oxoguanine glycosylase, and Nei-like DNA glycosylase 2 (NEIL2), in innate immunity (13, 16, 17, 18, 19). Our recent studies have demonstrated that 8-oxoG-bound 8-Oxoguanine glycosylase in the promoters of proinflammatory genes facilitates NF-κB recruitment to target gene promoters and enhances their expression (20, 21). In contrast, we found that mice deficient in NEIL2 are highly susceptible to tumor necrosis factor α (TNFα)- or lipopolysaccharide-mediated neutrophil recruitment in mice lungs (17); however, NEIL2's role as a modulator of innate immunity is not fully understood.

The canonical NF-κB activation pathway, primarily in response to proinflammatory cytokines such as TNFα, regulates the expression of various genes, including cytokines, chemokines, and cell adhesion molecules (21, 22). Aberrant NF-κB activation and chemokine/cytokine expression are not only associated with pathogenesis of several inflammatory diseases, such as multiple sclerosis, rheumatoid arthritis, chronic obstructive pulmonary disease, and asthma (23, 24, 25, 26) but are also widely implicated in inflammation-induced neoplasia and cancer (27, 28, 29). A recent analysis of copy number variation using the data from COSMIC and TCGA databases revealed that NEIL2's level is generally low in most cancers (30, 31). We and others have also reported that functional variants of NEIL2 are risk factors for lung cancer, squamous cell carcinoma, and breast cancer in BRCA2 mutation carriers (32, 33, 34, 35). Although *Neil2*-null (*Neil2*^*−/−*^) mice are viable and do not show spontaneous tumor development (17, 36), their susceptibility to inflammatory stimuli prompted us to explore the mechanistic basis of NEIL2's role in innate immunity.

Here, we report that NEIL2 suppresses NF-κB–mediated proinflammatory gene expression. We also demonstrate that NEIL2 interacts with Rel homology region of RelA and blocks NF-κB's promoter occupancy. Importantly, we show that intranasal delivery of endotoxin free recombinant NEIL2 suppresses the NF-κB–mediated immune response in mouse lung. Thus, NEIL2-mediated inhibition of NF-κB signaling may provide a beneficial immunotherapeutic strategy for the prevention of chronic inflammatory human diseases.

## Results

### NEIL2 deficiency increases susceptibility to systemic inflammation

**Figure 1:**
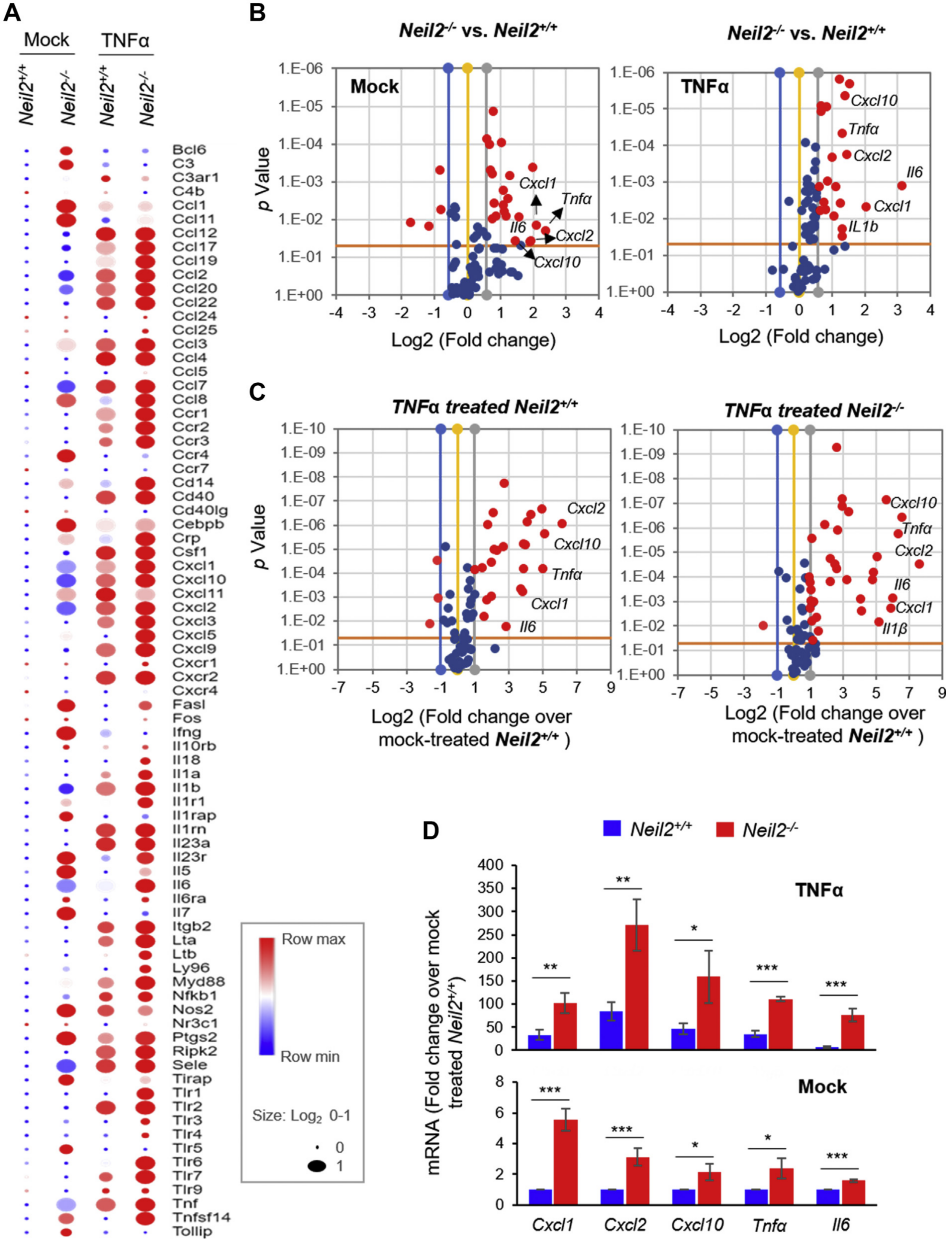
**Inflammatory gene expression in the lungs of *Neil2***^*−****/****−*^***versus Neil2***^***+/+***^**mice after systemic administration of TNFα.***A*, hierarchical clustering of expression of genes related to inflammatory chemokine/cytokines in mock or TNFα-treated (intraperitoneal) *Neil2*^*+/+*^ and *Neil2*^*−/−*^ mice lung relative to the mock-treated *Neil2*^*+/+*^ group. The color bar indicates the log2-fold change in the transcript level; *red* and *blue colors* indicate high and low expression levels, respectively. *B*, volcano plots of mRNA expression of proinflammatory genes in *Neil2*^*−/−*^*versus Neil2*^*+/+*^ mice lung, post mock- (*left*) or TNFα-treatment (*right*). *C*, volcano plots of proinflammatory mRNA expression in TNFα-treated *Neil2*^*+/+*^ (*left*) or TNFα-treated *Neil2*^*−/−*^ (*right*) mice lung over the mock-treated *Neil2*^*+/+*^ group. *B* and *C*, *x*-axis, log2-fold change; *y*-axis, *p* value; the *red dots* depict differentially expressed genes with a *p* value < 0.05 and a fold change >1.5 between the two groups. *D*, validation of multiplex array data for expression of indicated genes by real-time quantitative reverse transcription polymerase chain reaction in mock- (*lower panel*) or TNFα-treated (*upper panel*) *Neil2*^*+/+*^ or *Neil2*^*−/−*^ mice lung relative to mock-treated *Neil2*^*+/+*^ mice lung. Results are normalized to *Gapdh*. Error bars represent ±standard deviation from the mean. *A–D*, statistical analysis: n = 3 independent experiments from samples prepared by pooling lungs from n = 3 mice per group. ∗*p* < 0.05; ∗∗*p* < 0.01; ∗∗∗*p* < 0.005 *versus Neil2*^*+/+*^ groups. NEIL2, Nei-like DNA glycosylase 2; TNFα, tumor necrosis factor α.

In an effort to understand the role of NEIL2 in modulating inflammatory gene expression, we induced systemic inflammation in *Neil2*-null (*Neil2*^*−/−*^) and WT (*Neil2*^*+/+*^) mice *via* intraperitoneal administration of a single dose of TNFα or phosphate buffered saline (PBS) as mock and conducted a multiplex Real-Time qPCR (RT-qPCR) analysis of 79 inflammation-associated genes (Fig. 1*A*). Mock- or TNFα-treated *Neil2*^*−/−*^ mice lungs presented over 1.5-fold increased expression of ~20 genes (*p* < 0.05) compared with *Neil2*^*+/+*^ mice (Fig. 1*B*). Compared with mock-treated *Neil2*^*+/+*^, TNFα treatment increased expression of 25 inflammatory genes over 2-fold (*p* < 0.05) in *Neil2*^*+/+*^ mice (Fig. 1*C*, left); however, following TNFα treatment, 48 genes were upregulated more than a 2-fold (*p* < 0.05) in *Neil2*^*−/−*^ mice (Fig. 1*C*, right). TNFα-treated *Neil2*^*−/−*^ lungs presented increased expression of genes involved in neutrophil trafficking (*Cxcl1*and *Cxcl2*), the Th1 response, natural killer cells, or monocyte trafficking (*Cxcl10, Ccl3,* and *ccl2*), as well as in the regulation of immune response, hematopoiesis, or inflammation (*Tnfα*, *Il6,* and *IL1β*) causing inflammation of lungs. The multiplex gene array data were further validated using RT-qPCR (list of primers; Table S1) in lungs (Fig. 1*D*) and in two additional tissues, brain and muscle (Fig. S1, *A* and *B*) with similar results, indicating a systemic inflammatory response. Collectively, these data imply that NEIL2 deficiency alone results in a higher basal level of systemic inflammation that can be aggravated further on exposure to an external inflammatory stimulus.

### NEIL2 suppresses NF-κB–mediated inflammatory responses in mice lung

**Figure 2:**
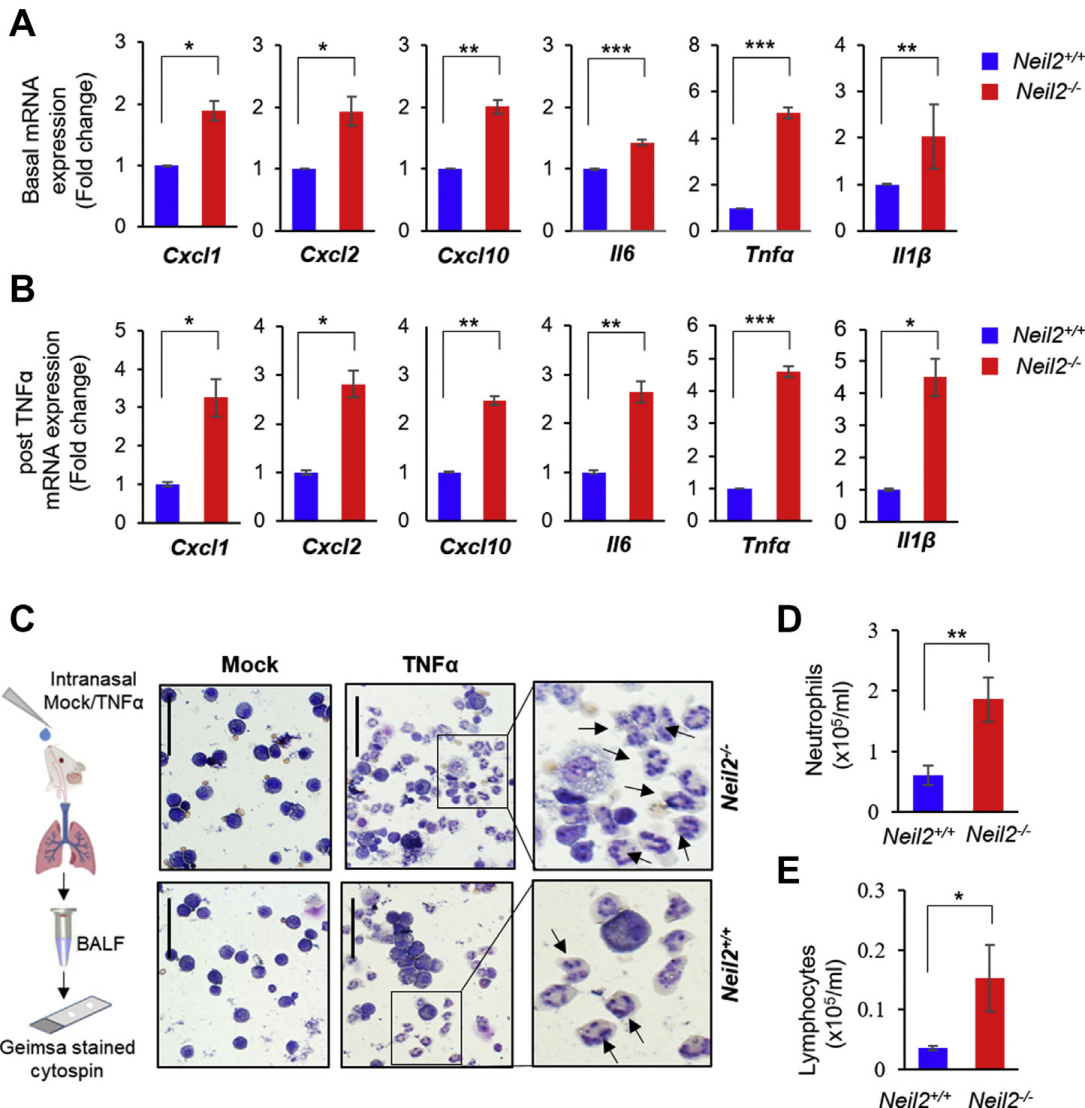
**TNFα-induced inflammation and inflammatory gene expression in the lungs of *Neil2***^*−****/****−*^***versus Neil2***^***+/+***^**mice.***A* and *B*, real-time quantitative reverse transcription polymerase chain reaction of mRNA expression of *Cxcl1, Cxcl2, Cxcl10, Il6, Tnfα,* and *Il1β,* in *Neil2*^*+/+*^ or *Neil2*^*−/−*^ mice lung post intranasal (i.n.) treatment with mock (*A*) or TNFα, represented as fold change in area under the curve (AUC) of mRNA expression over time course of 15 to 120 min posttreatment (*B*). Results are normalized to *Gapdh* and represented as fold change over mock treated *Neil2*^*+/+*^ groups shown in *A*. *C*, representative images of GIEMSA-stained cytospin preparations of cells in broncho-alveolar lavage fluid (BALF) collected from *Neil2*^*+/+*^ or *Neil2*^*−/−*^ groups, 16 h post mock or TNFα (i.n.) treatment. Scale bar: 50 μm. Digital magnification (6×) of TNFα-treated samples are shown in the far-right panels; arrows point to neutrophils. *D* and *E*, analysis of neutrophil (*D*) and lymphocyte (*E*) count in BALF from *Neil2*^*+/+*^ or *Neil2*^*−/−*^ male mice lungs collected 16 h post TNFα treatment (i.n.). All error bars represent ± standard deviation from the mean. (*A* and *B*) n = 2 independent experiments, with data collected from n = 3 or 4 mice per group; (*C–E*) n = 6 mice per group. ∗*p* < 0.05; ∗∗*p* < 0.01; ∗∗∗*p* < 0.005 *versus Neil2*^*+/+*^ groups. NEIL2, Nei-like DNA glycosylase 2; TNFα, tumor necrosis factor α.

Under pathophysiological conditions, neutrophils are recruited to the lungs in response to chemokines, expression of which are induced because of infections and/or physical or chemical injuries (37). Therefore, to better understand the physiological role of NEIL2 in modulating lung inflammation, we delivered TNFα or PBS (as mock) to *Neil2*^*+/+*^ or *Neil2*^*−/−*^ mice *via* intranasal (i.n.) route and analyzed the expression of proinflammatory genes in the lungs over a period of 120 min by RT-qPCR. The basal level of *Cxcl1, Cxcl2*, *Cxcl10, Il6, Tnfα,* and *ILβ* expression in *Neil2*^*−/−*^ mice lung was significantly higher compared with *Neil2*^*+/+*^ (Fig. 2*A*). TNFα treatment resulted in further increase in expression of all the genes tested, especially in *Neil2*^*−/−*^ compared with *Neil2*^*+/+*^ mice lungs (Figs. 2*B* and S2). CXC chemokines are strong chemoattractants, and their enhanced expression plays a critical role in modulating neutrophil and lymphocyte migration (38, 39). Neutrophil and lymphocyte levels in bronchoalveolar lavage fluid (BALF) of mock-treated mice were low (Fig. 2*C*, far left panel). However, following TNFα treatment, *Neil2*^*−/−*^ mice showed significantly higher neutrophil (~3-fold; *p* = 0.0052) (Fig. 2*C*, middle and far right panels, and Fig. 2*D*) recruitment in the BALF compared with *Neil2*^*+/+*^ mice. These data are consistent with our earlier report (17). Additionally, we also observed a significantly higher number of lymphocytes (~4-fold; *p* = 0.02) in TNFα-treated lungs (Fig. 2*E*). These data are in accordance with the results observed with systemic inflammation in *Neil2*^*−/−*^ *versus Neil2*^*+/+*^ mice (Fig. 1) and further confirm that NEIL2-deficient mice not only has higher basal inflammatory state but also experience a greater insult from proinflammatory mediators following exposure to inflammatory agents.

### NEIL2 modulates NF-κB's promoter occupancy of proinflammatory genes

**Figure 3:**
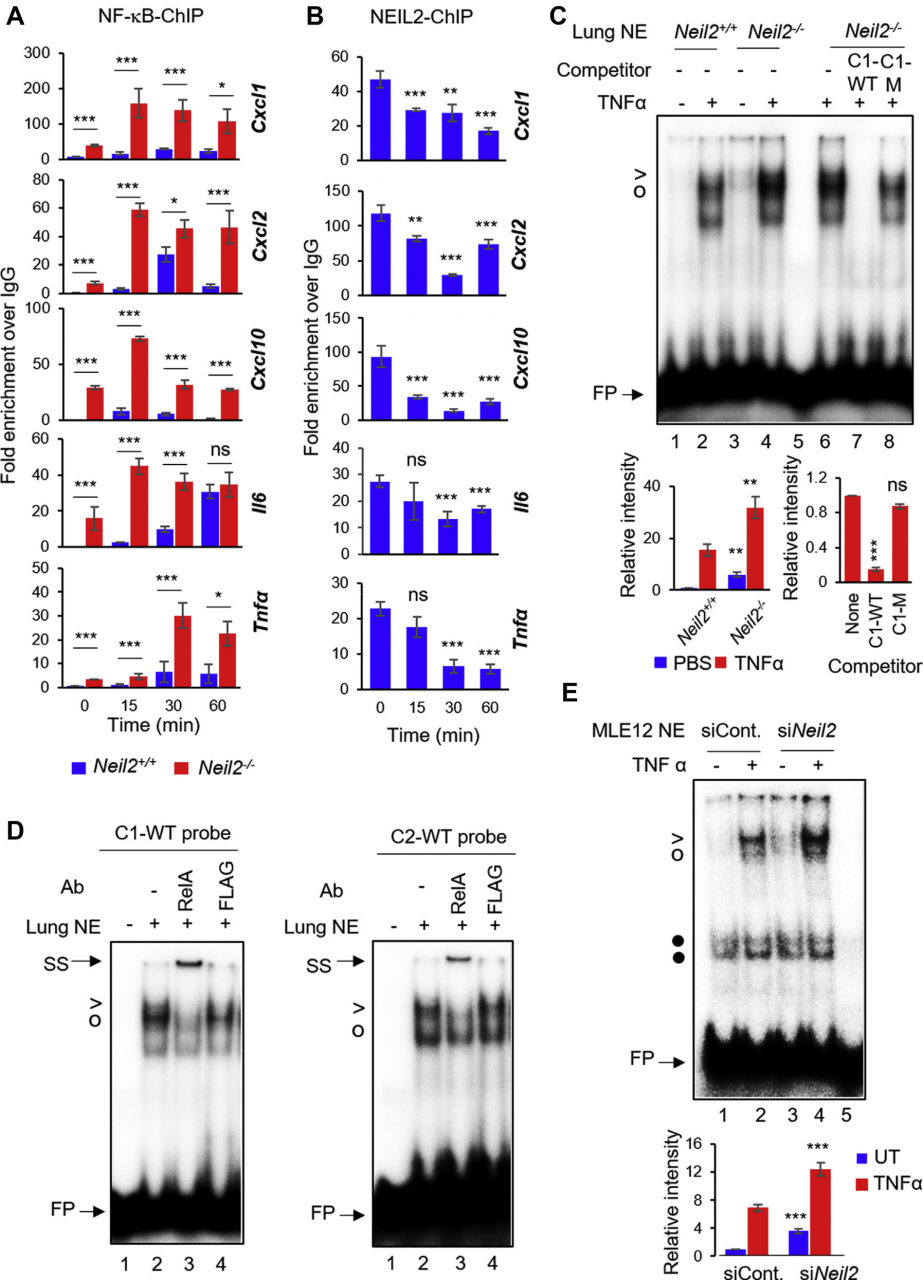
**Effect of NEIL2 on NF-κB's binding to its cognate motif derived from target gene promoters.***A* and *B*, chromatin immunoprecipitation quantitative polymerase chain reaction (ChIP-qPCR) of NF-κB/RelA binding at the promoter region of indicated genes in *Neil2*^*−/−*^*versus Neil2*^*+/+*^ mice lungs (*A*) or NEIL2 binding at the same loci in *Neil2*^*+/+*^ mice lung (*B*), post intranasal (i.n.) TNFα treatment for the indicated time points. Specific binding was calculated as the percent of input DNA and displayed as fold-enrichment over Immunoglobulin G (IgG). *C*, electrophoretic mobility-shift assay (EMSA) using nuclear extracts (NEs) from mock- or TNFα-treated (i.n.) *Neil2*^*+/+*^ or *Neil2*^*−/−*^ male mice lung (pooled, n = 3) with ^32^P-labeled probe containing WT κB-motif from mouse *Cxcl1* promoter (C1-WT); competition analysis (lanes 6–8) with 100-fold molar excess of WT (C1-WT) or mutant (C1-M) competitor; lane 5, no protein. > and ^O^ denote RelA-DNA complexes; • denotes nonspecific band; FP represents free probe. Histograms depict RelA-DNA band intensities from three independent EMSAs. *D*, super-shift analysis of NEs from TNFα-treated *Neil2*^*−/−*^ mice lung with C1-WT (*left panel*) or C2-WT (*right panel*) probes, using anti -RelA or -FLAG antibody (Ab). SS indicates band super shift; lane 1, no protein. *E*, EMSA of NEs from untreated (−) or TNFα-treated (+) NEIL2-deficient (si*Neil2*) or control (siCont.) MLE12 cells with C2-WT probe; lane 5, no protein. *C–E*, representative data images are shown from three independent experiments (n = 3). All error bars, ± standard deviation from the mean. ∗*p* < 0.05; ∗∗*p* < 0.01; ∗∗∗*p* < 0.005 *versus Neil2*^*+/+*^ groups for *A* and *C-left*, *versus* mock-treated (0 min) group for *B*, and no-competition lanes for *C-right*. NEIL2, Nei-like DNA glycosylase 2; TNFα, tumor necrosis factor α.

NF-κB is directly involved in the transcriptional regulation of a variety of chemokines/cytokines (40, 41, 42). Thus, we analyzed NF-κB/RelA occupancy at proinflammatory gene promoters by chromatin immunoprecipitation-qPCR (ChIP-qPCR, List of primers in Table S2). We observed a significantly higher enrichment of NF-κB/RelA at the promoter sites of *Cxcl1, Cxcl2*, *Cxcl10, Il6,* and *Tnfα* in *Neil2*^*−/−*^ mice lungs compared with *Neil2*^*+/+*^ groups (Fig. 3*A*). Intriguingly, a peak NEIL2 occupancy was also detected at the promoter sites of all the genes tested in mock-treated groups (Fig. 3*B*, 0 min); however, such binding of NEIL2 decreased significantly over time following TNFα treatment with the concurrent increase in NF-κB occupancy at the same loci (Fig. 3*B versus* A). The inverse nature of NEIL2 and NF-κB occupancy at proinflammatory gene promoters suggests an interplay between NEIL2 and NF-κB at promoter sites and may explain the NEIL2-mediated modulation of inflammatory genes. To test whether NEIL2 modulates NF-κB's binding to its cognate motif, we analyzed NF-κB binding by electrophoretic mobility-shift assay (EMSA) using the nuclear extracts (NEs) from PBS/TNFα-treated *Neil2*^*+/+*^ or *Neil2*^*−/−*^ mice lungs with radiolabeled oligonucleotides (oligos) containing the NF-κB DNA response element or κB-motif present in the promoters of *Cxcl1, Cxcl2,* and *Il6*. *Neil2*^*−/−*^ mice displayed a significantly higher level of NF-κB-DNA complex in both mock (-) and TNFα-treated (+) groups with the WT-*Cxcl1* oligos (Fig. 3*C*, lane 3 *versus* 1 and lane 4 *versus* 2, respectively) and -*Cxcl2* or *-Il6* oligos (Fig. S3, *A* and *C*, lane 3 *versus* 1 and lane 4 *versus* 2, respectively). The NF-κB–DNA complex was competed out effectively by a 100-fold molar excess of unlabeled C1-WT, C2-WT, and IL6-WT, but not by a mutant -κB oligo C1-M, C2-M, and IL6-M (Fig. 3*C*, lane 8 *versus* 7, Fig. S3*B*, lane 3 *versus* 2 and Fig. S3*C*, lane 8 *versus* 7, respectively). The mutant oligos lacked binding with recombinant RelA (Fig. S3*D*), confirming the specificity of the binding with WT κB-motif. The addition of anti-RelA antibody to the NEs caused a shift of the protein–DNA complex band with both C1-WT and C2-WT oligos, confirming the identity of RelA in the complex (Fig. 3*D*). Furthermore, siRNA-mediated NEIL2 depletion in mouse lung epithelial (MLE12) cells (Fig. S3*E*) also resulted in higher NF-κB–DNA binding compared with NEIL2-proficient cells, with or without TNFα-treatment (Fig. 3*E*, lane 3 *versus* 1 and lane 4 *versus* 2). All these data strongly suggest that NEIL2 modulates NF-κB's promoter binding activity and subsequent gene expression, and provides an opportunity to therapeutically modulate the detrimental effects of inflammation.

### NEIL2 suppresses promoter occupancy of NF-κB via association with DNA binding domain of RelA

**Figure 4:**
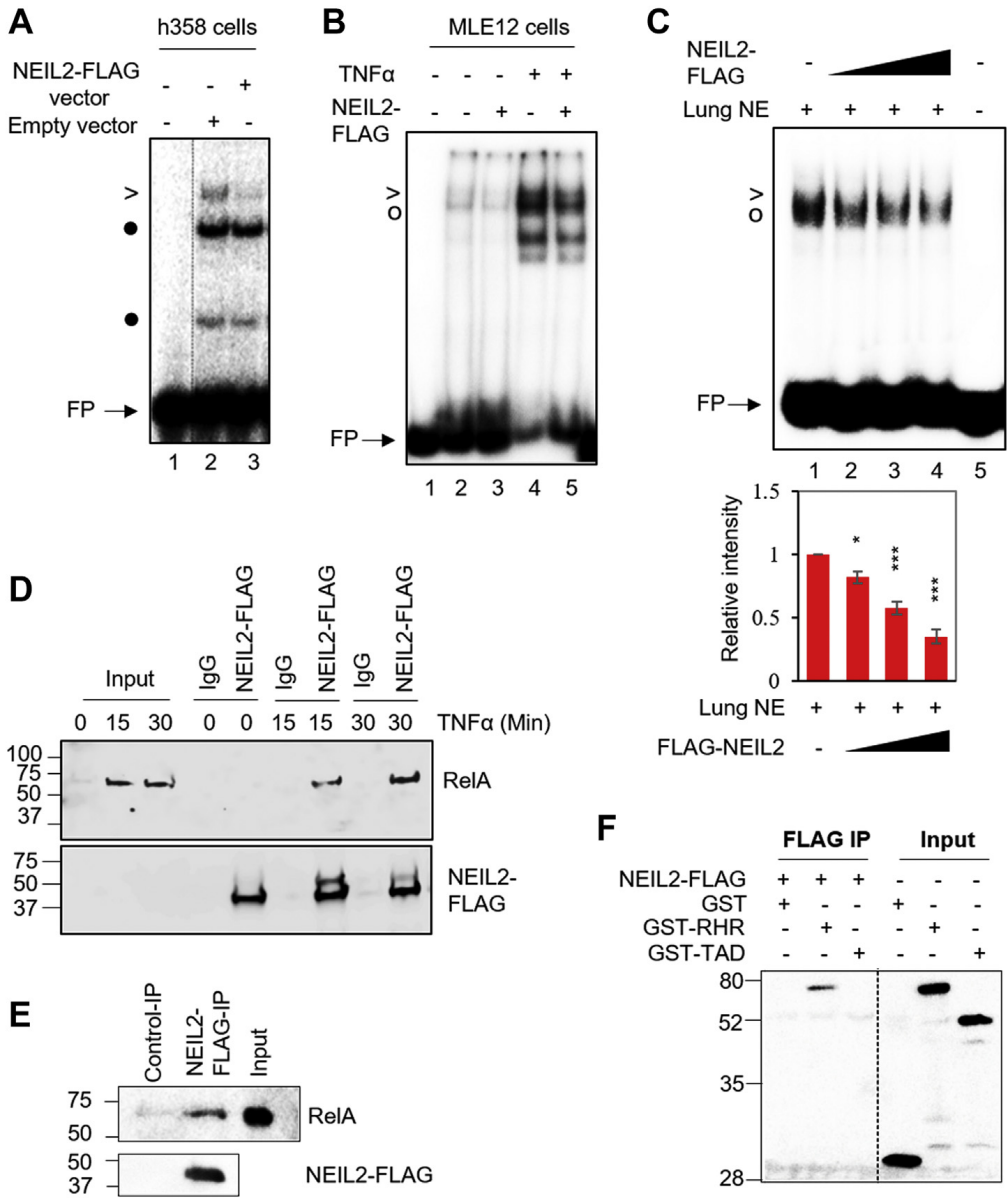
**Effects of purified NEIL2 on NF-κB's binding to its cognate motif *in vitro*.***A* and *B*, electrophoretic mobility-shift assays (EMSAs) of NEs from h358 cells (*A*) or untreated (−) or TNFα-treated (+) MLE12 cells (*B*) expressing Empty (−) or NEIL2-FLAG (+) vectors with C1-WT probe; Lane 1, no protein. *C*, EMSA of NE from TNFα-treated mice lung (2 μg) added to NEIL2-FLAG (5, 10, 20 nM, lanes 2–4) with ^32^P-labeled C1-WT probe in the binding reaction. Histograms depict relative RelA-DNA band intensities from three independent EMSAs. >and ^O^ denote RelA-DNA complexes; • denotes nonspecific band; FP represents free probe. *D*, co-immunoprecipitation (Co-IP) of RelA using anti-FLAG beads incubated with NEs of BEAS2B cells stably expressing NEIL2-FLAG. *E* and *F*, Co-IP analysis of the interaction between recombinant full-length His-tagged-RelA (*E*) or indicated GST-tagged-RelA active domains (*F*) and NEIL2-FLAG, immunoprecipitated using anti-FLAG beads preincubated with whole cell lysates of h358 cells transfected with control or FLAG-NEIL2 vectors. Immunoblot analysis of Co-IPs was performed with antibodies against RelA or FLAG (*D* and *E*) and GST (*F*). Input represents total cell lysate. Representative data images are shown from three independent experiments (n = 3). All error bars, ± standard deviation from the mean. ∗*p* < 0.05; ∗∗*p* < 0.01; ∗∗∗*p* < 0.005 *versus* no-competition lane. NE, nuclear extract; NEIL2, Nei-like DNA glycosylase 2; TNFα, tumor necrosis factor α.

To investigate the mechanism by which NEIL2 acts as a negative modulator of NF-κB, we first tested whether overexpression of NEIL2 directly inhibits NF-κB's DNA binding*.* Ectopically expressed C-terminal FLAG-tagged NEIL2 (NEIL2-FLAG) in human lung 358 (h358) cells where NF-κB is constitutively active and is present in the nucleus (43) or MLE12 cells (Fig. S4, *A* and *B*) significantly decreased NF-κB-DNA complex formation at κB-site in both the cell lines compared with cells transfected with empty-vector (Fig. 4*A*, lane 3 *versus* 2 and Fig. 4*B*, lane 3 *versus* 2 and 5 *versus* 4). Next, we performed EMSA with the NEs prepared from TNFα-treated mouse lungs and found that addition of cell purified NEIL2 significantly blocked formation of the NF-κB-DNA complex in a dose dependent manner (Fig. 4*C*, lanes 2–4 *versus* 1). To test whether NEIL2-mediated inhibition of the NF-κB-motif binding is due to protein–protein interaction, we immunoprecipitated NEIL2 using the anti-FLAG antibody from the human bronchial epithelial cell line BEAS-2B stably expressing NEIL2-FLAG and detected RelA in the immunocomplex as early as 15 min following TNFα treatment (Fig. 4*D*). Purified NEIL2-FLAG from h358 cells also pulled down recombinant RelA (Fig. 4*E*). Furthermore, purified NEIL2-FLAG specifically co-immunoprecipitated the DNA-binding Rel homology region (RHR) of RelA (GST-RHR), but not the transcription-activation domain of RelA (GST-TAD) (Fig. 4*F*), suggesting that NEIL2 inhibits the RelA:DNA complex formation by interacting with DNA-binding domain of RelA. Thus, it is conceivable that NEIL2's association with the DNA binding domain of RelA prevents RelA from binding to the κB site and consequently suppresses the proinflammatory gene expression.

### Purified NEIL2 suppresses the TNFα-induced inflammatory response *in vivo*

**Figure 5:**
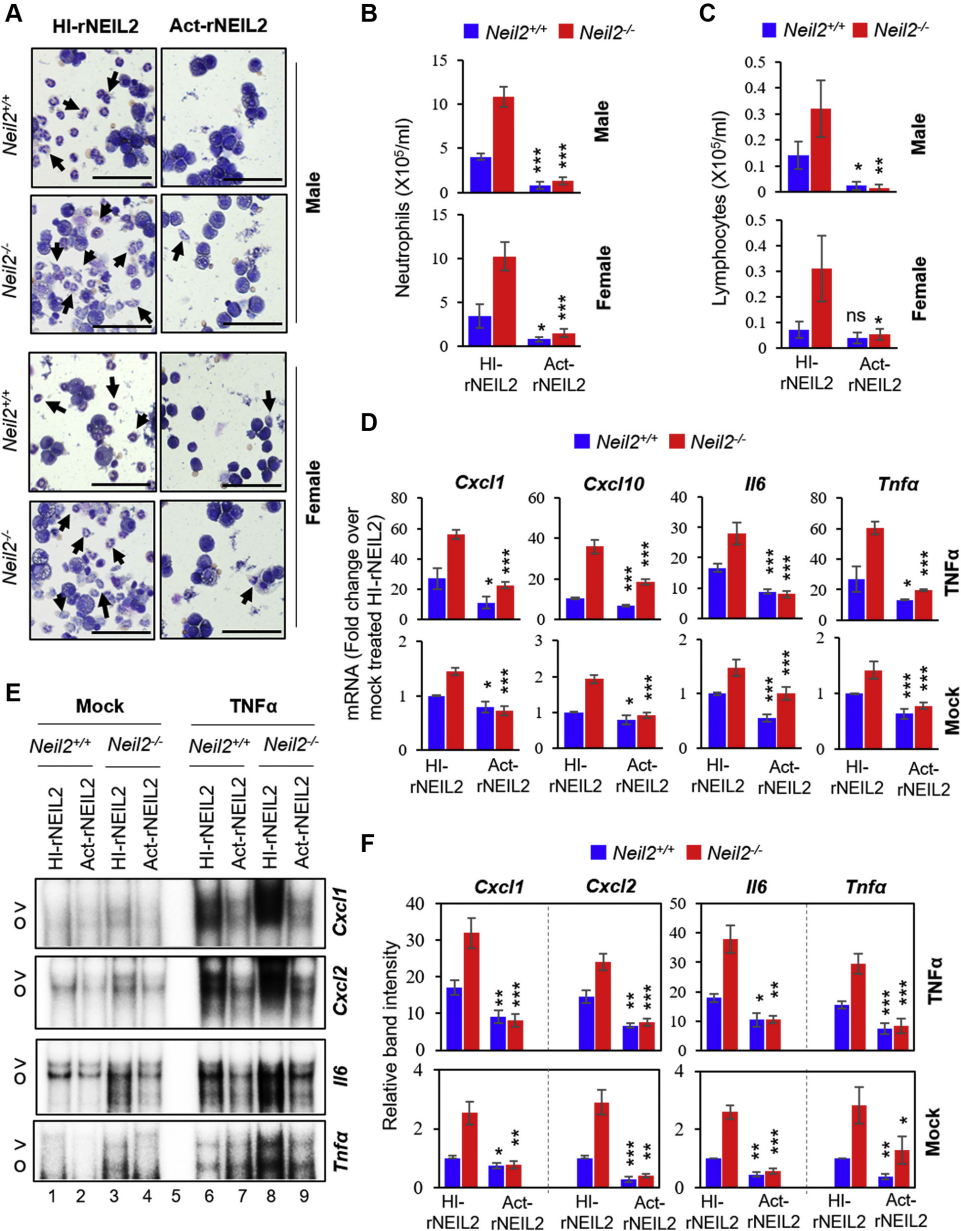
**Effects of intranasal (i.n.) delivery of purified NEIL2 on TNFα-mediated inflammation in mouse lung.***A–C*, analysis of differential cell populations in broncho-alveolar lavage fluid (BALF) collected 16 h post TNFα-treatment (i.n.) from *Neil2*^*+/+*^ or *Neil2*^*−/−*^ male and female mice lungs transduced with HI-rNEIL2 or Act-rNEIL2 (i.n., 72 h). Representative images of GEIMSA-stained cytospin preparations of cells recovered from BALF (A), Scale bar: 50 μm, arrows indicate neutrophils; histograms represent the number of neutrophils (*B*) and lymphocytes (*C*). *D*, real-time quantitative reverse transcription polymerase chain reaction analysis of mRNA expression of *Cxcl1, Cxcl2, Il6,* and *Tnfα* in mock- or TNFα-treated (i.n., 1 h) *Neil2*^*+/+*^ or *Neil2*^*−/−*^ mice transduced with HI-rNEIL2 or Act-rNEIL2 (i.n., 72 h). *E* and *F*, representative electrophoretic mobility-shift assay images (*E*) and quantitation from three independent experiments (*F*) of NF-κB binding in NEs from mock- or TNFα-treated (i.n.) *Neil2*^*+/+*^ or *Neil2*^*−/−*^ male mice lungs transduced with HI-rNEIL2 or Act-rNEIL2 (i.n.), using ^32^P-labeled WT probes for *Cxcl1, Cxcl2, Il6* and *Tnfα* κB-sites as indicated; (*E*) lane 5, no protein. The full size gel images for panel *E* are displayed in Figure S6. All error bars represent ±standard deviation from the mean. (*A–C*) n = 3 mice per group, (*D*) n = 4 mice per group and (*F*) n = 3 independent experiments, performed from pooled samples of mouse lungs (n = 3 mice per group). ns, not significant; ∗*p* < 0.05; ∗∗*p* < 0.01; ∗∗∗*p* < 0.005 *versus* HI-rNEIL2 transduced group. NEs, nuclear extracts; NEIL2, Nei-like DNA glycosylase 2; TNFα, tumor necrosis factor α.

Given the antiinflammatory properties of NEIL2, we attempted to evaluate its potential as a therapeutic biologic for inflammatory diseases. We delivered an optimized amount of endotoxin-free His-tagged recombinant NEIL2 (rNEIL2, *E.coli* purified) *via* the intranasal route to the lungs of age- and sex-matched *Neil2*^*+/+*^ and *Neil2*^*−/−*^ mice using a synthetic peptide, K16SP, as a carrier (44). Mice were then treated with TNFα, 72 h post-NEIL2 treatment, and lungs were collected for differential cell count, RT-qPCR, and NE preparations. The enzymatically inactive heat-inactivated rNEIL2 (HI-rNEIL2) (Fig. S5*A*) was used as a control. HI-rNEIL2 and active rNEIL2 (Act-rNEIL2) were uniformly delivered to male/female mouse lungs (Fig. S5, *B* and *C*). Act-rNEIL2 blocked neutrophil count in BALF, 16 h post mock (Fig. S5*D*) and TNFα treatment (Fig. 5, *A* and *B*) in both *Neil2*^*+/+*^ and *Neil2*^*−/−*^ male or female mice compared with HI-rNEIL2 transduced groups. Similarly, Act-rNEIL2 decreased the recruitment of TNFα-induced lymphocytes (Fig. 5*C*) in both *Neil2*^*+/+*^ and *Neil2*^*−/−*^ groups. Chemokine/cytokine expression was significantly suppressed in Act-rNEIL2 recipient mouse lungs, 1 h post mock or TNFα treatment as analyzed by RT-qPCR (Fig. 5*D*). Furthermore, a remarkable decrease in NF-κB-DNA complex formation was observed in Act-rNEIL2 transduced *Neil2*^*+/+*^ and *Neil2*^*−/−*^ male mice lung NEs compared with HI-rNEIL2-transduced groups, as analyzed by EMSA using *Cxcl1*, *Cxcl2, Il6,* or *Tnfα* oligo probes (Figs. 5*E* and S6, lanes 2, 4, 7 and 9 *versus* 1, 3, 6 and 8, respectively, and Fig. 5*F*). Similar results were observed with the NEs prepared from Act-NEIL2 transduced *Neil2*^*+/+*^ and *Neil2*^*−/−*^ female mice lungs for all tested probes (Fig. S7). Collectively, these data provide strong evidence for the therapeutic potential of NEIL2 to actively block NF-κB–mediated inflammatory responses by directly suppressing NF-κB's binding to its consensus motifs within the target gene promoters.

## Discussion

Persistent activation of inflammatory molecules plays a central role in many chronic human diseases. Atypical NF-κB signaling underlies the uncoordinated expression of prototypical proinflammatory cytokines which promotes the pathogenesis of inflammatory diseases (23, 24, 45) and remains a target for therapeutic intervention. In this report, we describe the noncanonical role of NEIL2 as a repressor of NF-κB that has the potential to mitigate complications of hyperinflammation associated with various human pathologies including chronic inflammatory diseases and cancer.

We have demonstrated that a subset of the genes encoding proinflammatory cytokines such as TNFα and IL6 that regulate hematopoiesis and innate immune response, chemokines such as the CCL and CXCL family members that regulate migration of monocytes, neutrophils, and natural killer cells were expressed at higher levels in mock- or TNFα-treated *Neil2*^*−/−*^ compared with WT mice lungs, primarily because of increased NF-κB occupancy at the promoter region of those genes. Most intriguingly, NEIL2 occupies the same promoter loci as NF-κB under normal physiologic condition; however, NEIL2's binding is decreased significantly, but not entirely, with a concomitant increase in NF-κB's promoter occupancy following TNFα stimulus. Thus, an interplay exists between NEIL2 and NF-κB at the promoter sites where NEIL2 blocks NF-κB's binding to the κB-motif under unstimulated conditions. Furthermore, NEIL2 was found to directly interact with the DNA-binding domain of RelA, a component of the NF-κB dimer, and prevent its binding to the promoter. As reported earlier, DNA binding of the NF-κB subunit RelA is regulated by a variety of cofactors, such as ribosomal subunit S3, nucleoplasmin-1, and Sam68, among others (46, 47, 48, 49, 50), that directly bind to the RHR of RelA. While these cofactors support DNA binding of RelA, retinoblastoma protein, on the other hand, binds to the RHR of RelA and blocks it from binding to the promoters of some genes, such as programmed death ligand-1 (51). NEIL2 thus may adopt a similar mechanism to inhibit formation of the RelA:DNA complex. This observation clearly explains the increased expression of NF-κB–dependent inflammatory chemokines/cytokines in the absence of NEIL2.

To validate the antiinflammatory role of NEIL2 *in vivo*, we developed a method of noninvasive and stable delivery of recombinant NEIL2 to mice lungs. The primary reason for focusing on the lung as a model for inflammation studies is that, unlike other internal organs, the lungs are continuously exposed to various types of endogenous and exogenous inflammatory stimuli (52, 53). Furthermore, intranasal administration is a noninvasive route for drug delivery, especially for protein-based therapeutic molecules (54, 55, 56). Intranasally transduced endotoxin-free rNEIL2 not only decreased proinflammatory gene expression but also significantly blocked recruitment of neutrophils in both mock- and TNFα-treated *Neil2*^*−/−*^ and *Neil2*^*+/+*^ mice lungs primarily because of significantly reduced NF-κB–DNA complex formation at the promoter sites of proinflammatory genes in active NEIL2 transduced groups.

**Figure 6:**
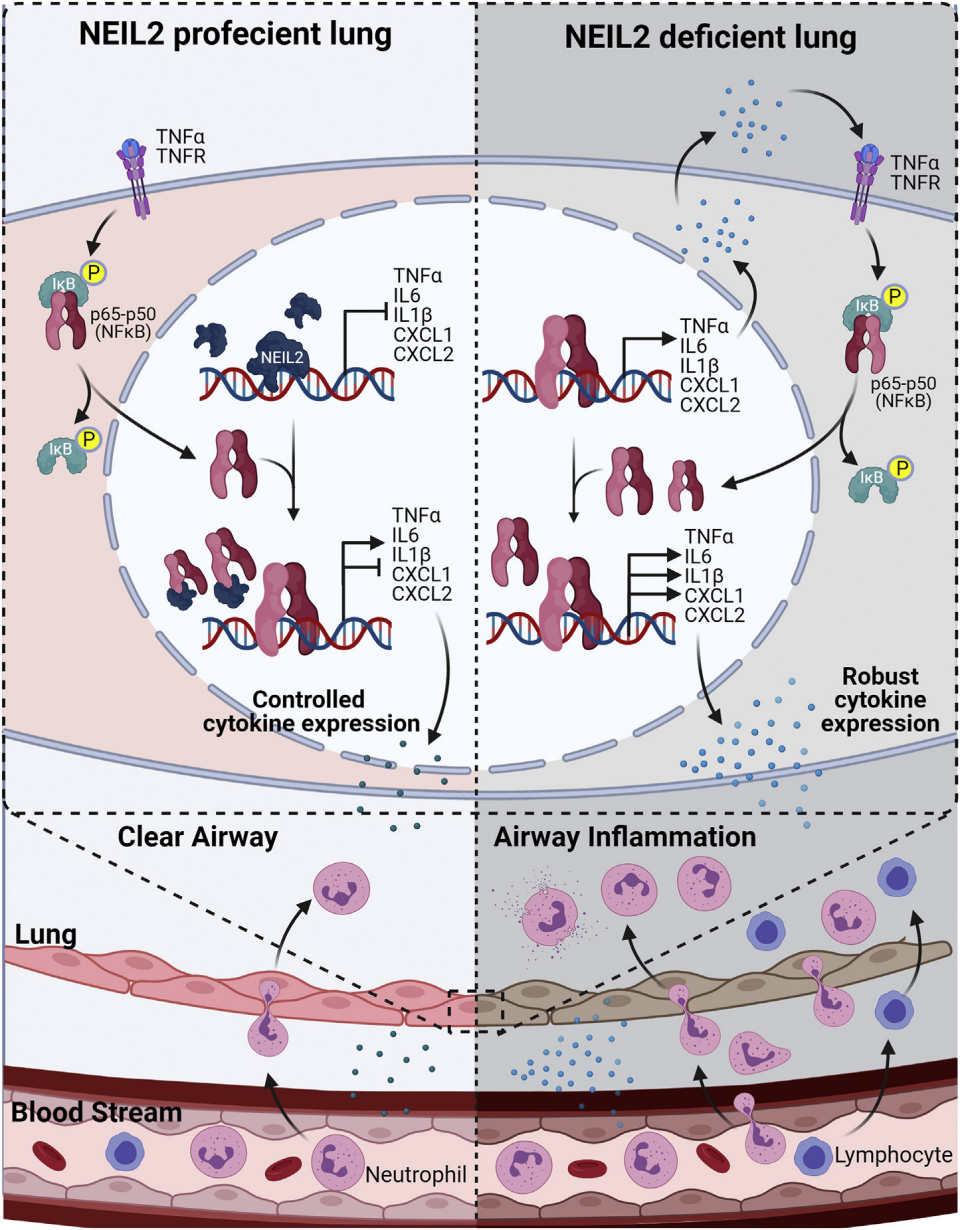
**A model depicting the role of NEIL2 in modulating NF-κB-mediated signaling in lung.** (Created with BioRender.com) IL, interleukin; NEIL2, Nei-like DNA glycosylase 2; TNFα, tumor necrosis factor α.

Based on those above observations, here we provide a model summarizing antiinflammatory aspect of NEIL2 (Fig. 6). In the absence of proinflammatory stimuli, NEIL2 binds to the promoter regions of various proinflammatory chemokines/cytokines preventing unwarranted binding of NF-κB to its cognate sites and thereby block expression of those gene. Following an external stimulus, activated NF-κB migrates to the nucleus and displaces NEIL2 from the promoters. Then, the released NEIL2 binds to the RHR of RelA and consequently blocks its access to the κB-site. NEIL2 thus participates in controlling the expression of proinflammatory chemokines and cytokines leading to normal neutrophil and lymphocyte recruitment to lung airways. However, in the absence of NEIL2, NF-κB can readily bind to proinflammatory gene promoters leading to increased basal gene expression that aggravates further following a proinflammatory stimulus. As a result, robust chemokine/cytokine expression leads to increased neutrophil and lymphocyte recruitment to the lung airways that may cause tissue damage. Hence, an interplay between NEIL2 and RelA would be essential for combating chronic activation of NF-κB and maintaining immune homeostasis.

It is well established that dysregulation of NF-κB contributes to the pathogenesis of chronic inflammatory diseases as well as cancer and thus remains a focus for therapeutic intervention. Antiinflammatory drugs are commonly used for treating various inflammatory diseases, but in many cases, such treatments are associated with serious side effects and thus cannot provide a long-term beneficial effect (57, 58). While these inhibitors are able to reduce inflammation, they are unable to repair the genome damage that cells have already incurred, and such persistent DNA lesions will continue to contribute to the pathogenesis. We thus propose that intrapulmonary delivery of NEIL2, with its dual role in regulating inflammation and DNA repair, has strong potential as a therapeutic agent, especially for chronic inflammatory conditions such as asthma, chronic obstructive pulmonary disease, and other human lung diseases.

## Experimental procedures

### Mice

The generation of *Neil2*^*−/−*^ mice on the C57BL/6J background was described previously (17). All animal breeding and experiments were conducted in accordance with the guidelines of the Institutional Animal Care and Use Committee and approved protocols (Hazra, protocol no. 0606029D, and Boldogh, protocol no. 0807044A). *Neil2*^*+/+*^ and *Neil2*^*−/−*^ mice (male and female) 8 to 10 weeks of age were used in the study. Mice were treated either intraperitoneally with PBS (mock) or 85 μg/kg TNFα (BioLegend, catalog no. 575202), or i.n. with PBS or 20 ng/lung TNFα, as described previously (17). In some cases, 10 μg of rNEIL2 (active or heat inactivated) was mixed with 10 μM carrier peptide K16SP and incubated for 30 min at 37 °C; then NEIL2-peptide mix was delivered to mice lung i.n. 72 h prior to mock or TNFα treatment. Animals were euthanized and tissues were collected for various experiments.

### Collection of broncho-alveolar lavage fluid and differential cell count

Sixteen hours after intrapulmonary delivery of PBS (as mock) or TNFα, mice were euthanized, and BALF was collected by cannulating the trachea and washing the lungs twice with 0.7-ml ice-cold 1× PBS. The BALF was centrifuged at 3000 rpm to pellet the cells. Cells were re-suspended and washed with 1× PBS, and the total cell number was calculated. Cytospin slides were prepared and stained with Wright-Giemsa for differential cell count. The numbers of neutrophils, lymphocytes, and macrophages were determined by microscopic examination of a minimum of 400 cells/slide on each stained cytospin slide (59, 60).

### Cell culture and various treatments

Mouse lung epithelial 12 (MLE 12, ATCC CRL-2110) cells, human 358 (h358, ATCC CRL-5807) cells, and NEIL2-FLAG expressing BEAS2B stable cells were grown at 37 °C and 5% CO_2_ in respective DMEM/F-12 (1:1) and RPMI 1640 medium containing 10% fetal bovine serum, 100 units/ml penicillin, and 100 units/ml streptomycin. For all experiments, 50 to 60% confluent cells were used. Cells were cultured in media without serum for 24 h and then kept untreated or treated with 20 ng/ml TNFα for different time points. We routinely test *mycoplasma* contaminations in all our cell lines using the PCR-based Venor GeM *Mycoplasma* Detection Kit (Sigma, catalog no. MP0025).

### Gene expression with real-time quantitative PCR

Total RNA extraction was performed from cells or lung tissues using TRIzol Reagent (Invitrogen, catalog no. 15596026). Genomic DNA was removed, and up to 2 μg RNA was used to synthesize cDNA using a PrimeScript RT Kit with gDNA Eraser (TaKaRa, catalog no. RR047A). qPCR was carried out using TB Green Premix Ex Taq II (Tli RNase H Plus; TaKaRa, catalog no. RR820A) in Applied Biosystems 7500 Real-Time PCR Systems with thermal cycling conditions of 94 °C for 5 min (94 °C for 10 s and 60 °C for 1 min) for 40 cycles, and 60 °C for 5 min. The target mRNA levels were normalized to that of *Gapdh*. Sequences for the qPCR primers used are listed in Table S1. In each case, DNase-treated RNA samples without reverse transcriptase were used to rule out genomic DNA contamination.

### Inflammatory response and autoimmunity RT^2^ profiler PCR array

A mouse cDNA expression array, inflammatory response, and autoimmunity RT^2^ Profiler PCR Array (Qiagen, catalog no. 330231 PAMM-077ZA), including 84 genes involved in autoimmune and inflammatory immune responses, was used per manufacturer's protocol. Total RNA was isolated, and cDNAs were prepared as described above. The cDNAs were then subjected to qPCR using RT^2^ Profiler PCR Array and TB Green Premix Ex Taq II (Tli RNase H Plus). Hierarchical clusters for 79 expressing genes were constructed with Morpheus (https://software.broadinstitute.org/morpheus/), and volcano plots were generated in MS Excel using mean fold change and *p* values calculated from three independent PCR arrays. (A complete list of the 84 genes included in this array is provided by Qiagen (at https://www.qiagen.com/us/shop/pcr/primer-sets/rt2-profiler-pcr-arrays/?catno=PAMM-077Z#geneglobe).

### Chromatin immunoprecipitation and quantitative PCR

ChIP assays were performed as described earlier (61, 62), with some modifications. Briefly, mock- or TNFα-treated mice tissues were chopped into small pieces before crosslinking in 1% formaldehyde for 10 min at room temperature, then homogenized in buffer containing 50 mM Tris-HCl pH 8.0, 10 mM EDTA, and 1% SDS with 1X protease inhibitor cocktail before sonication. The tissues then sonicated (Qsonica Sonicators) to an average DNA size of ~300 bp in 50 mM Tris-HCl pH 8.0, 10 mM EDTA, and 1% SDS with 1X protease inhibitor cocktail (Roche, catalog no. 4693132001). The supernatants were diluted with 15 mM Tris-HCl pH 8.0, 1.0 mM EDTA, 150 mM NaCl, 1% Triton X-100, 0.01% SDS, and protease inhibitors, and incubated with ChIP grade anti-p65 or RelA (NF-κB, Santa Cruz, catalog no. sc-372) or NEIL2 (in house) (17) antibodies overnight at 4 °C. Immunocomplexes were captured by Protein A/G PLUS agarose beads (Santa Cruz, catalog no. sc-2003) that were then washed sequentially in buffer I (20 mM Tris-HCl pH 8.0, 150 mM NaCl, 1 mM EDTA, 1% Triton-X-100 and 0.1% SDS); buffer II (same as buffer I, except containing 500 mM NaCl); buffer III (1% NP-40, 1% sodium deoxycholate, 10 mM Tris-HCl pH 8.0, 1 mM EDTA); and finally with 1X Tris-EDTA (pH 8.0) buffer at 4 °C for 5 min each. The immunocomplexes were extracted from the beads with elution buffer (1% SDS and 100 mM NaHCO_3_) and de-crosslinked for 4 h at 65 °C. DNA was isolated by phenol-chloroform extraction and ethanol precipitation using GlycoBlue (Life Technologies, catalog no. AM9516) as carrier. ChIP samples were analyzed by qPCR using specific primers (Table S1), and data are represented as percent of input DNA normalized to IgG.

### Immunoblot analysis

Whole cell extracts (25 μg) or NEs (50 μg) were electrophoresed using a 4 to 12% Mini-PROTEAN TGX Gel (BioRad, catalog no. 456-1094), then transferred to PVDF membranes (BioRad, catalog no. 162-0175). After blocking in 5% skimmed milk in TBST buffer, the membranes were probed with anti-NEIL2 (developed in house), anti-FLAG (Sigma, catalog no. F1804), anti-His (GeneTex, catalog no. GTX628914), anti-RelA (p65) (Santa Cruz, catalog no. sc-372), or anti-GST (Biobharati, Catalog no. AB0020) antibodies. Anti-GAPDH (GeneTex, catalog no. GTX100118), anti-HDAC2 (GeneTex, catalog no. GTX109642), or anti-tubulin (Biobharati, Catalog no. BB-AB0119) antibodies were used as loading controls. In each case, the blot was stripped using Restore Plus stripping buffer (Thermo Scientific, catalog no. 46430) and re-probed with other antibodies.

### Gene knockdown by siRNA transfection

MLE12 cells were transfected with 80 nM siRNAs specific to NEIL2 (Sigma, catalog no. SASI_Mm01_00048613) or Mission universal control siRNA (Sigma, catalog no. SIC001) using Lipofectamine 2000 (Invitrogen, catalog no. 11668027) twice on consecutive days, according to the supplier's protocol. Cells were then rested for 48 h and used for subsequent analysis.

### Transient transfection of cells

MLE12 cells or h358 cells at approximately 50% confluency were transiently transfected with vector expressing FLAG-tagged NEIL2 (NEIL2-FLAG vector) (4 μg) or empty vector as control (4 μg) using Lipofectamine 2000, according to the supplier's protocol. To monitor transfection efficiency, a reporter gene construct (0.25 μg) containing β-galactosidase downstream to the SV40 promoter was co-transfected. Cells were allowed to recover for 16 h in media with serum and then were incubated with serum-free medium for 24 h before TNFα treatment (20 ng/ml). Total RNA, whole cell lysates, and NEs were prepared for subsequent RT-qPCR, immunoblot analysis, or EMSAs, respectively.

### Protein expression and purification

Recombinant FLAG-tagged-NEIL2 (NEIL2-FLAG) was purified from h358 cells. A 10 cm dish of h358 cells was transfected with a mammalian expression vector pcDNA3 containing C-terminal-FLAG-tagged-Neil2. Next day, cells were harvested in ice-cold 1X PBS. After centrifugation, the cellular pellet was resuspended in lysis buffer (25 mM Tris-HCl pH 7.5, 150 mM NaCl, 5% glycerol, 0.1% NP-40, 0.2 mM EDTA, 0.5 mM phenylmethylsulfonyl fluoride (PMSF), 1X protease inhibitor cocktail, 1 mM DTT) and sonicated. After centrifugation, preequilibrated (with lysis buffer) anti-Flag beads (Sigma, catalog no. A2220) were added and mixed by rotation for 2 h at 4 °C. The beads were then washed four times with lysis buffer, and bound protein was eluted with elution buffer (lysis buffer +0.1 mg/ml 3XFlag peptide). Peak fractions were concentrated with Centriprep 30-kDa and was stored at −80 °C in aliquots. WT recombinant His-tagged NEIL2 (rNEIL2) was purified from *E. Coli* using protocol as described earlier (63). Briefly, pET22b (Novagen) vector containing C-terminal His-tagged-WT NEIL2 coding DNA sequence was transformed into *E.coli BL21(DE3)* RIPL Codon-plus cells. Log-phase culture (A_600_ = 0.4–0.6) of *E. coli* was induced with 0.5 mM isopropyl-1-thio-β-D-galactopyranoside at 16 °C for 16 h. After centrifugation, the cell pellets were suspended in a lysis buffer (buffer A) containing 25 mM Tris-HCl, pH 7.5, 500 mM NaCl, 10% glycerol, 1 mM β-mercaptoethanol (β-ME), 0.25% Tween 20, 5 mM imidazole, 2 mM PMSF. After sonication, the lysates were spun down at 13,000 rpm, and the supernatant was loaded onto HisPur Cobalt Superflow Agarose (Thermo Scientific, catalog no. 25228) previously equilibrated with buffer A and incubated for 2 h at 4 °C. After washing with buffer A with gradient increasing concentration of imidazole (10, 20, 30, 40 mM), the His-tagged proteins were eluted with an imidazole gradient (80–500 mM imidazole in buffer containing 25 mM Tris-HCl, pH = 7.5, 300 mM NaCl, 10% glycerol, 1 mM ß-ME, 0.25% Tween 20). After elution, the peak protein fractions were dialyzed against buffer C (1XPBS, pH 7.5, 1 mM DTT, and 25% glycerol). EndoTrap red 5/1 (LIONEX GmbH catalog no. LET0002) columns were used to remove endotoxin from the protein preparations. The endotoxin level of the protein was measured using Pierce LAL Chromogenic Endotoxin Quantitation Kit (Thermo Scientific, catalog no. 88282). Protein with endotoxin levels less than 0.001 EU/μg protein was used in *in vivo* experiments.

Recombinant His-RelA (rHis-RelA) full-length baculovirus construct was kindly provided by Dr James Kadonaga (64). Sf9 suspension cultures were infected with rHis-RelA full-length baculovirus at a cell density of 1 × 10^6^/ml and allowed to grow for 60 h postinfection. The cells were harvested and lysed in lysis buffer (10 mM Tris-HCl pH 7.5, 500 mM NaCl, 0.1% Nonidet P-40, 10% glycerol, 15 mM imidazole, 10 mM β-ME, 2 mM PMSF, and protease inhibitor mixture) by sonication. The lysate was clarified by filtering with 0.22 μm and mixed with slurry of nickel-nitrilotriacetic acid resin (Ni-NTA Agarose; Qiagen, catalog no. 30210) in batch at 4 °C for 3 h. The resin was thoroughly washed with lysis buffer containing 30 mM imidazole and 300 mM NaCl before elution using the same buffer with 400 mM imidazole and 200 mM NaCl. Elution was done twice at 4 °C for 30 and 10 min, respectively. Both elutions were pooled and dialyzed three times 1 h each. First and second dialysis were done against dialysis buffer 1 (10 mM Tris-HCl, pH 7.5, 200 mM NaCl, 10 mM β-ME, 10% glycerol), and the third dialysis was against dialysis buffer 2 (10 mM Tris-HCl, pH 7.5, 200 mM NaCl, 10 mM β-ME, 5% glycerol). Protein was concentrated by centrifugation using Centriprep 30-kDa cutoff membrane concentrator unit (Millipore, catalog no. 4306) and loaded onto preparative Superdex 200 size exclusion column connected to an AKTA purifier (GE Healthcare) equilibrated with buffer containing 10 mM Tris-HCl pH 7.5, 200 mM NaCl, 10 mM DTT, 5% glycerol at room temperature. Peak fractions were concentrated again with Centriprep 30-kDa. Protein was stored at −80 °C in aliquots.

GST-tagged proteins (64), GST-RelA-AD were expressed in *E. coli Rosetta* cells by growing cells harboring the expression plasmid (pGEX-4T containing RelA 429–551) to A_600_ 0.4 followed by induction with 0.1 mM IPTG overnight at room temperature. The fusion protein was purified in a single step using a glutathione-Sepharose column (gift from Biobharati Life Science) from the crude cell lysate (150 mM NaCl, 25 mM Tris-HCl pH 7.5, 10% glycerol) followed by elution with 10 mM glutathione. All the purified preparations of proteins were stored in aliquots at −20 °C.

### Preparation of nuclear extracts

NEs were prepared from lung tissues and cells as described previously (65), with slight modifications. Briefly, tissues were homogenized to obtain single cell suspension in buffer containing 15 mM Tris-HCl pH 7.9, 0.25 M sucrose, 60 mM KCl, 15 mM NaCl, 5 mM EDTA, 0.5 mM Spermidine, 1 mM DTT, 0.1 mM PMSF, and 1X protease inhibitor cocktail and centrifuged at 3000 rpm for 5 min. The cell pellets were suspended in buffer A (25 mM Hepes pH 7.9, 5 mM KCl, 0.5 mM MgCl_2_, 1 mM DTT and 1X protease inhibitor cocktail) and centrifuged at 3000 rpm at 4 °C for 5 min. The cell pellets were re-suspended in buffer A with 0.5% Nonidet P-40, incubated on ice for 5 min and then centrifuged at 2500 rpm for 2 min at 4 °C. The pellets were washed with buffer A with 0.5% Nonidet P-40 at 2500 rpm for 2 min at 4 °C. The nuclear pellets were re-suspended in five volumes of buffer B (25 mM Hepes pH 7.9, 10% glycerol, 1 mM DTT, 0.1% Nonidet P-40, 0.1 mM PMSF and 1X protease inhibitors cocktail) and centrifuged at 2500 rpm for 2 min. The pelleted nuclei were lysed in buffer B supplemented with 350 mM NaCl by incubating on ice for 1 h with intermittent tapping. Homogenates were then centrifuged at 10,000 rpm at 4 °C for 15 min, and aliquots of supernatants were used immediately or stored at −80 °C.

### Electrophoretic mobility-shift assay

Sequences of the oligonucleotide probes used for EMSAs are listed in Table S2. The sense and antisense strands were annealed, and 5 pmol of annealed oligos were end-labeled with [γ-^32^P] ATP (PerkinElmer, catalog no. BLU502A250UC) using T4-polynucleotide kinase (New England BioLabs, catalog no. M0201). Unincorporated nucleotides were removed by Micro Bio-Spin P-6 Gel Columns (BioRad, catalog no. 7326221). For DNA-protein interaction, 15 to 25 fmol of ^32^P-labeled oligonucleotide probes were incubated with NE (2–4 μg) or purified proteins (5–2000 nM) in buffer containing 0.5 μg of poly dI.dC. (Thermo Scientific, catalog no. 20148E), 2 to 10 μg of acetylated BSA (Promega, catalog no. R3961), 10 mM Tris-HCl pH 7.9, 50 mM NaCl, 1 mM MgCl2, 1 mM EDTA, 5 mM DTT, and 5% glycerol, for 10 min at room temperature in a total volume of 6 to 20 μl. The reaction mixture was then subjected to electrophoresis (200 V in 0.3X Tris-buffered EDTA solution at 4 °C) using 5% nondenaturing polyacrylamide gels. Gels were fixed in acetone: methanol: H2O (10:50:40) solution for 15 min, exposed to a Phosphor screen for 12 to 16 h and scanned using Typhoon Phosphorimager (GE Healthcare). For competition experiments, a 100- to 500-fold molar excess of unlabeled, WT, or mutant duplex oligonucleotides were added to the binding reaction. For super shift analysis, 2 μl of anti- RelA or FLAG antibody was added to the binding reaction before addition of the ^32^P-labeled probes.

### Analysis of DNA glycosylase activity of NEIL2

DNA glycosylase/AP lyase activity of purified active or heat inactivated NEIL2 was tested using a 5-OHU containing 5′ ^32^P-labeled duplex oligo substrate, as described earlier (66, 67). Briefly, a 51-mer oligo 5′-GCT TAG CTT GGA ATC GTA TCA TGT AXA CTC GTG TGC CGT GTA GAC CGT GCC-3′, with X indicating 5-OHU, was first labeled at the 5′-terminus using T4 polynucleotide kinase and [γ^32^P]ATP and then annealed with the complementary strand (with G opposite 5-OHU) to produce an 11 nt bubble in the middle. The labeled oligo duplex was incubated with 25 to 50 ng of Act-rNEIL2 or HI-rNEIL2 at 37 °C for 20 min in 50 mM Hepes pH 8.0, 50 mM KCl, 100 μg/ml BSA, and 5% glycerol. The reaction was stopped with 80% formamide in 10 mM NaOH, and the cleaved DNA was separated by denaturing gel electrophoresis in 15% polyacrylamide containing 7 M urea in 90 mM Tris-borate pH 8 and 2 mM EDTA. Gels were exposed to a Phosphor screen for 3 to 4 h and scanned using Typhoon Phosphorimager.

### Co-immunoprecipitation

h358 cells expressing control or NEIL2-Flag vector were lysed in IP buffer (25 mM Tris-HCl pH 7.5, 150 mM NaCl, 1% Triton X-100, 5% glycerol, 1 mM DTT, 0.5 mM PMSF, and 1X protease inhibitor cocktail) and incubated with preequilibrated anti-FLAG beads for 1 h at 4 °C with rotation. Beads were then washed with IP buffer and resuspended in 300 μl of IP buffer containing 1 μg of rHis-RelA or GST-tagged-RelA active domains and incubated for 1 h at 4 °C with rotation. Beads were then washed in IP buffer, and the pull-down complex was subject to immunoblot analysis using anti-RelA, FLAG, or GST antibodies.

### Statistical analysis

Two-sided unpaired Student's *t* test (http://www.ruf.rice.edu/∼bioslabs/tools/stats/ttest.html) and online MedCalc statistical software (https://www.medcalc.org/calc/comparison_of_means.php) were used for analysis of statistical significance between two sets of data. Number of independent experiments denotes the number of biological replicates. Significance was evaluated at level *p* > 0.05 (not significant), *p* < 0.05 (∗), *p* < 0.01 (∗∗), and *p* < 0.005 (∗∗∗), as the case may be.

## Data availability

All data that support the findings of this study are contained within the article and its supporting information.

## Supporting information

This article contains supporting information.

## Conflict of interest

The authors declare that they have no conflicts of interest with the contents of this article.
