# Supplementary material for: Intrapulmonary administration of purified NEIL2 abrogates NF-κB–mediated inflammation
Source: J Biol Chem. 2021 Apr 28;296:100723. doi: 10.1016/j.jbc.2021.100723 (PMC8164026; doi:10.1016/j.jbc.2021.100723)
Supplement: Figures S1 to S7 and Tables S1 and S2 [file mmc1.pdf]

Intrapulmonary administration of purified NEIL2 abrogates NF-κB-mediated inflammation

Nisha Tapryal[1], Shandy Shahabi[2], Anirban Chakraborty[1], Koa Hosoki[1,3], Maki Wakamiya[4], Gobinda Sarkar[5,6], Gulshan Sharma[1], Victor J. Cardenas[1], Istvan Boldogh[7], Sanjiv Sur[1,3], Gourisankar Ghosh[2], and Tapas K. Hazra[1*]

List of Supporting Material

1. Table S1
2. Table S2
3. Figure S1-S7

Table S1. List of primers used in qPCR assays.

Exon Primers	Sequence
mCxcl1-exon-For<br>mCxcl1-exon-Rev	5′ CCTATCGCCAATGAGCTGC 3′<br>5′ ACTTGGGGACACCTTTTAGC 3′
mCxcl2-exon-For<br>mCxcl2-exon-Rev	5′ CACCAACCACCAGGCTACA 3′<br>5′ GGATGATTTTCTGAACCAGGGG 3′
mCxcl10-exon-For<br>mCxcl10-exon-Rev	5′ GATCCCTCTCGCAAGGACG 3′<br>5′ TCAACACGTGGGCAGGATAG 3′
mIl6-exon-For<br>mIl6-exon-Rev	5′ GTCCTTCCTACCCCAATTTCCA 3′<br>5′ AGGTTTGCCGAGTAGATCTCAA 3′
mTnfα-exon-For<br>mTnfα-exon-Rev	5′ AAATGGTTCGTACAGCAGCG 3′<br>5′ GCACGGAAGTAGTCTCCACG 3′
mIL1β-exon-For<br>mIL1β-exon-Rev	5′ CATCAGCACCTCACAAGCAG 3′<br>5′ TAAGTGGTTGCCCATCAGAGG 3′
mGapdh-exon-For<br>mGapdh-exon-Rev	5′ GTCACCAGGGCTGCCATTTG 3′<br>5′ GATGTTAGTGGGGTCTCGCT 3′
**Promoter Primers**	**Sequences**
mCxcl1-Pro-For<br>mCxcl1-Pro-Rev	5′ ATCCTTGGGAGTGGAGCAAG 3′<br>5′ GGAGTCTGGAGTGCTGGAAC 3′
mCxcl2-Pro-For<br>mCxcl2-Pro-Rev	5′ AGCGCAGACATCACTTCCTT 3′<br>5′ TGAAGTGTGGCTGGAGTCTG 3′
mCxcl10-Pro-For<br>mCxcl10-Pro-Rev	5′ GCTCACGCTTTGGAAAGTGAA 3′<br>5′ ATGTCTCTCAGCGGTGGATG 3′
mIl6-Pro-For<br>mIl6-Pro-Rev	5′ CCCCACCCTCCAACAAAGATT 3′<br>5′ CAGAGAGGAACTTCATAGCGGT 3′
mTNFα-Pro-For<br>mTNFα-Pro-Rev	5′ TCTCAAGCTGCTCTGCCTTC 3′<br>5′ GACCATGCCTGTGTCTATTTCC 3′

Table S2. List of the mouse DNA oligonucleotides used in EMSA analysis. For clarity, the NF-κB motif sequences are shown in red and bases marked in blue represent the alterations in sequence to generate mutation.

DNA Oligos	Label	Duplex Sequence
mCxcl1-WT	C1-WT (Cxcl1)	NF-κB Motif<br>5′ GAAACACCCTGTACTCCGGGAATTTCCCTGGCCCGGAGCTCTG 3′<br>3′ ATTTGTGGGACATGAGGCCCTTAAAGGGACCGGGCCTCGAGAC 5′
mCxcl1-Mutant	C1-M	5′ GAAACACCCTGTACTCCGAAAAGGTCCCTGGCCCGGAGCTCTG 3′<br>3′ CTTTGTGGGACATGAGGCTTTTCCAGGGACCGGGGCTCGAGAC 5′
mCxcl2-WT	C2-WT (Cxcl2)	NF-κB Motif<br>5′ GGACCCTGAGCTCAGGGAATTTCCCTGGTCCCCGGGCT 3′<br>3′ CCTGGGACTCGAGTCCCTTAAAGGGACCAGGGGCCCGA 5′
mCxcl2-Mutant	C2-M	5′ GGACCCTGAGCTCAGAAAAGGTCCCTGGTCCACGGGCT 3′<br>3′ CCTGGGACTCGAGTCTTTTCCAGGGACCAGGTGCCCGA 5′
mIl6-WT	IL6-WT (Il6)	NF-κB Motif<br>5′ TTTTTATCAAATGTGGGATTTTCCCATGAGTCTCAAAA 3′<br>3′ AAAAATAGTTTACACCCTAAAAGGGTACTCAGAGTTTT 5′
mIl6-Mutant	IL6-M	5′ TTGCTATTAAATGTGTTCGTTTGTACAGAGTCTCAAAA 3′<br>3′ AACGATAATTTACACAAGCAAACATGTCTCAGAGTTTT 5′
mTnfα-WT	Tnfα	NF-κB Motif<br>5′ AAGAACTCAAACAGGGGGCTTTCCCTCCTCAATATCAT 3′<br>3′ TTCTTGAGTTTGTCCCCCGAAAGGGAGGAGTTATAGTA 5′

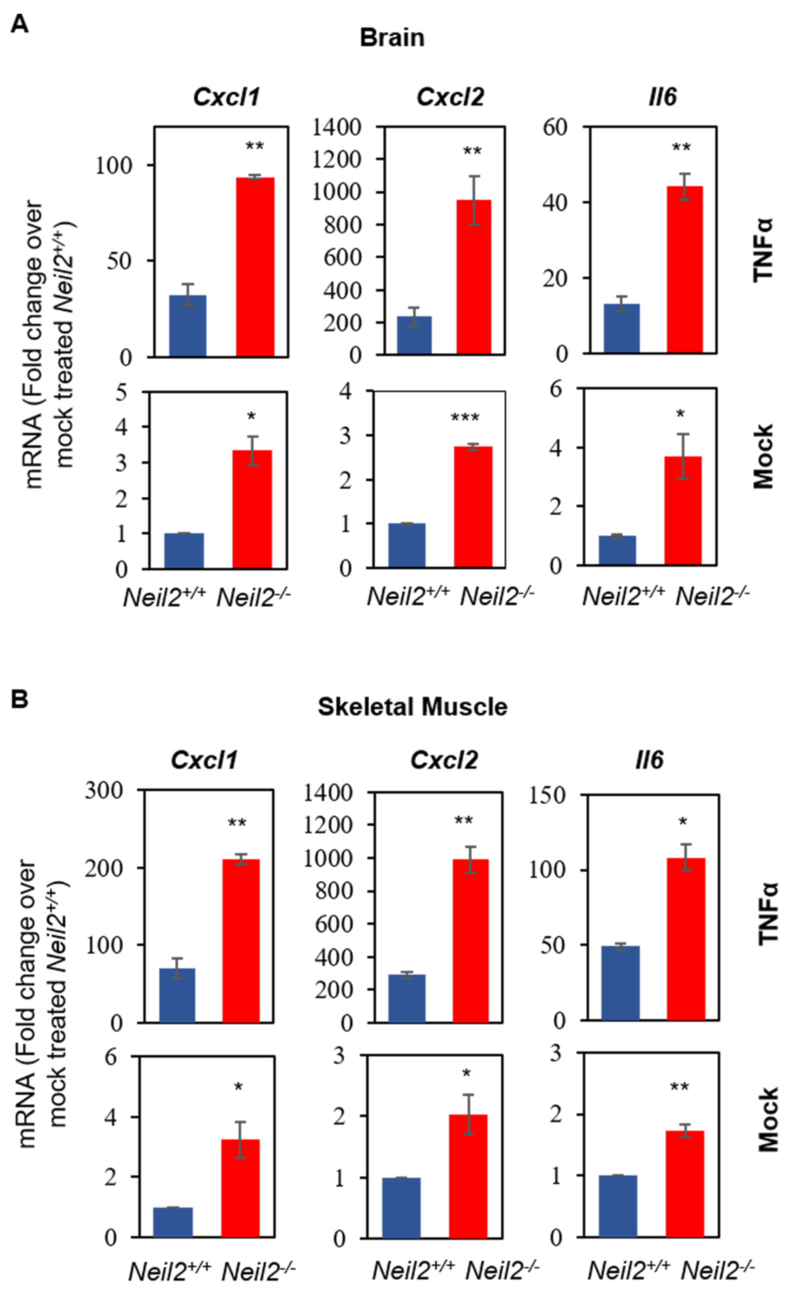

Figure. S1. Systemic inflammation in brain and skeletal muscle of *Neil2*$^{-/-}$ vs. *Neil2*$^{+/+}$ mice.
(**A** and **B**) Real-Time quantitative reverse transcription polymerase chain reaction analysis with total RNA isolated from brain (A) and skeletal muscles (B) of mock- (lower panel) or TNFα-treated (upper panel) (intraperitoneal) *Neil2*$^{+/+}$ or *Neil2*$^{-/-}$ mice relative to mock-treated *Neil2*$^{+/+}$ groups. Results are normalized to *Gapdh*. Error bars represent ± standard deviation from the mean. n=2 independent experiments from samples prepared by pooling tissues from n=3 mice per group. *=P<0.05; **=P<0.01; ***=P<0.005 vs. *Neil2*$^{+/+}$ groups.

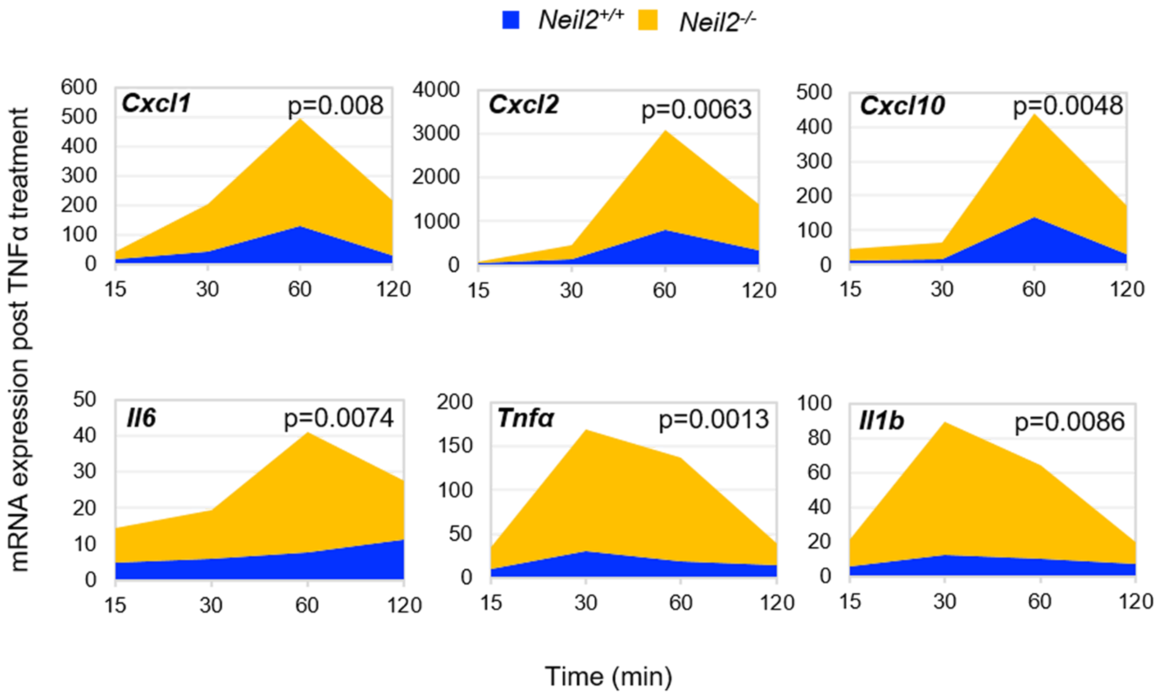

Figure. S2. TNFα-induced inflammatory gene expression in the lungs of *Neil2*$^{-/-}$ vs. *Neil2*$^{+/+}$ mice. Area plot displays mRNA expression of *Cxcl1*, *Cxcl2*, *Cxcl10, Il6, Tnfα* and *Il1β* in *Neil2*$^{+/+}$ or *Neil2*$^{-/-}$ mice lung at 15-, 30-, 60- and 120-min post TNFα intranasal treatment as analyzed by Real-Time quantitative reverse transcription polymerase chain reaction analysis. Results are normalized to *Gapdh* expression and mock-treated *Neil2*$^{+/+}$ groups; n=2 independent experiments, with data collected from n=4 mice per group; p-values for *Neil2*$^{-/-}$ vs. *Neil2*$^{+/+}$ groups are shown.

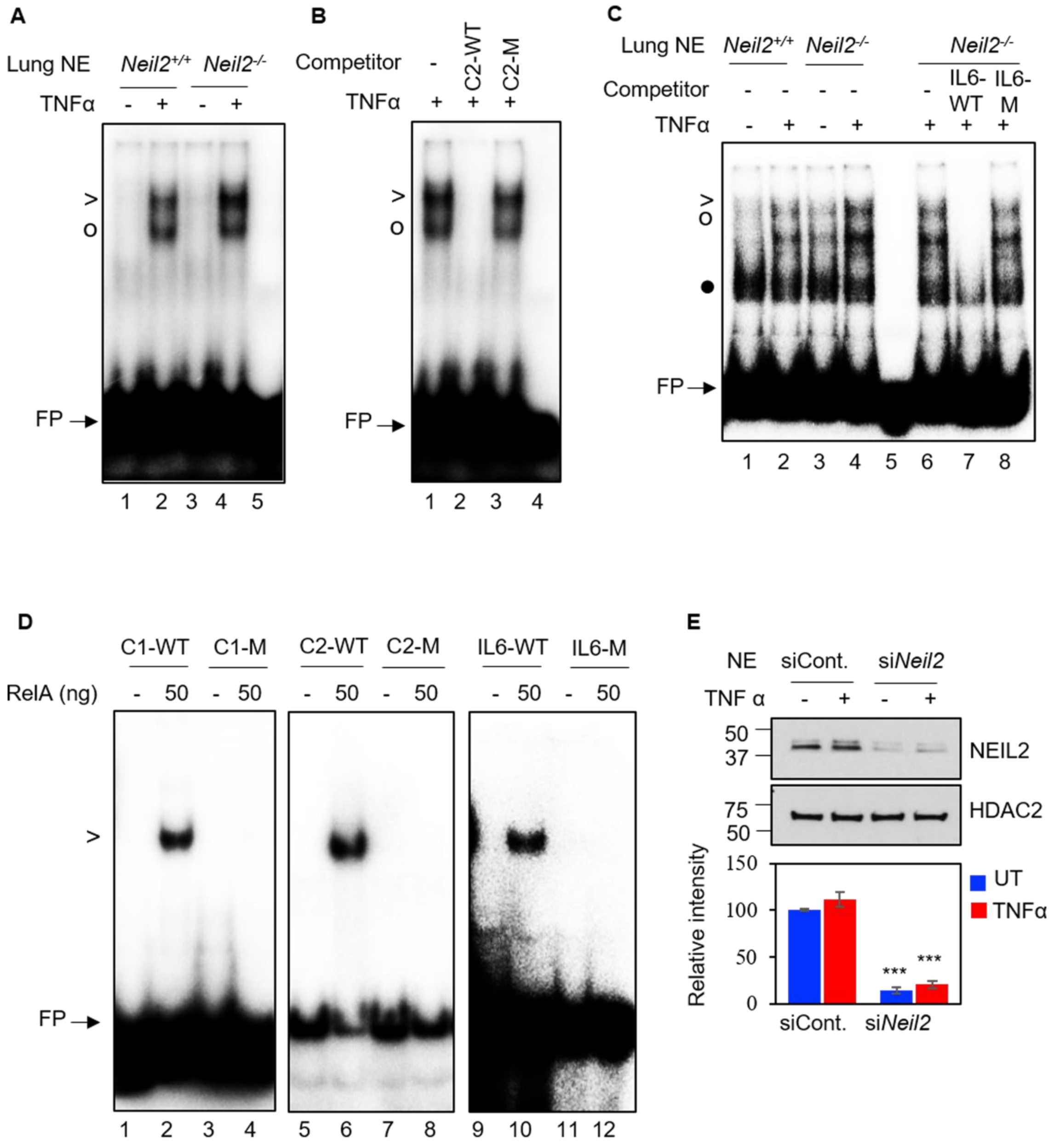

Figure. S3. Electrophoretic mobility-shift assays (EMSAs) of NF-κB's binding to its cognate motif derived from target gene promoters.

(**A**-**C**) EMSA using nuclear extracts (NEs) from mock- or TNFα-treated intranasally *Neil2*$^{+/+}$ or *Neil2*$^{-/-}$ male mice lung (pooled, n=3) with ^{32}P-labeled probe containing wild-type κB-motif from mouse *Cxcl2*- (C2-WT, A and B) and *Il6*- promoter (IL6-WT, C); competition analysis in B, lanes 1-3, and in C, lanes 6-8 with 100-fold molar excess of wild-type or mutant competitor as indicated. (**D**) EMSAs of recombinant RelA protein complex using ^{32}P-labeled wild-type C1-WT, C2-WT and IL6-WT or mutant C1-M, C2-M and IL6-M NF-κB-motifs; Lanes 1, 3, 5, 7, 9 and 11 show 'probe only' lanes. (**E**)

Immunoblot of NEIL2 in nuclear extracts (NEs) from untreated (-) or TNFα-treated (+) MLE12 cells transfected with control siRNA (si*Cont.*) or NEIL2-specific siRNA (si*Neil2*). HDAC2 was used as loading control; Quantification shows NEIL2 band intensities normalized to HDAC2 and represented relative to untreated *siCont.* group arbitrarily taken as 100. Representative data images from three independent experiments are shown. > and ^{O} denote RelA-DNA complexes; ● denotes nonspecific complex; FP represents free probe. Error bars represent ± standard deviation from the mean. n=3 independent experiments. ***=*P*<0.005 vs. *siCont.* groups.

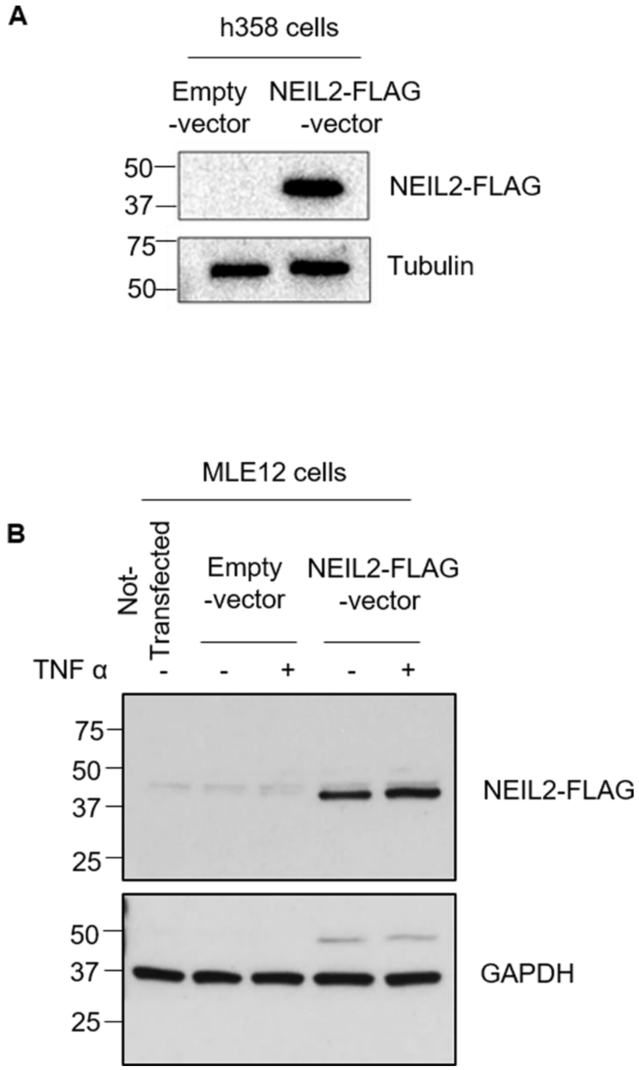

Figure. S4. NEIL2 overexpression in human and mouse cell lines.

(**A**) Immunoblot of cell extracts from h358 cells expressing control (Empty)- or NEIL2-FLAG-vectors, using anti -FLAG or -Tubulin antibodies. (**B**) Immunoblot of cell extracts from untreated (-) or TNFα-treated (+) MLE12 cells transfected with control or NEIL2-FLAG vectors, using anti -FLAG or -GAPDH antibodies. Un-transfected/untreated MLE12 cell extract was loaded as negative control. Representative data images from two independent experiments are shown.

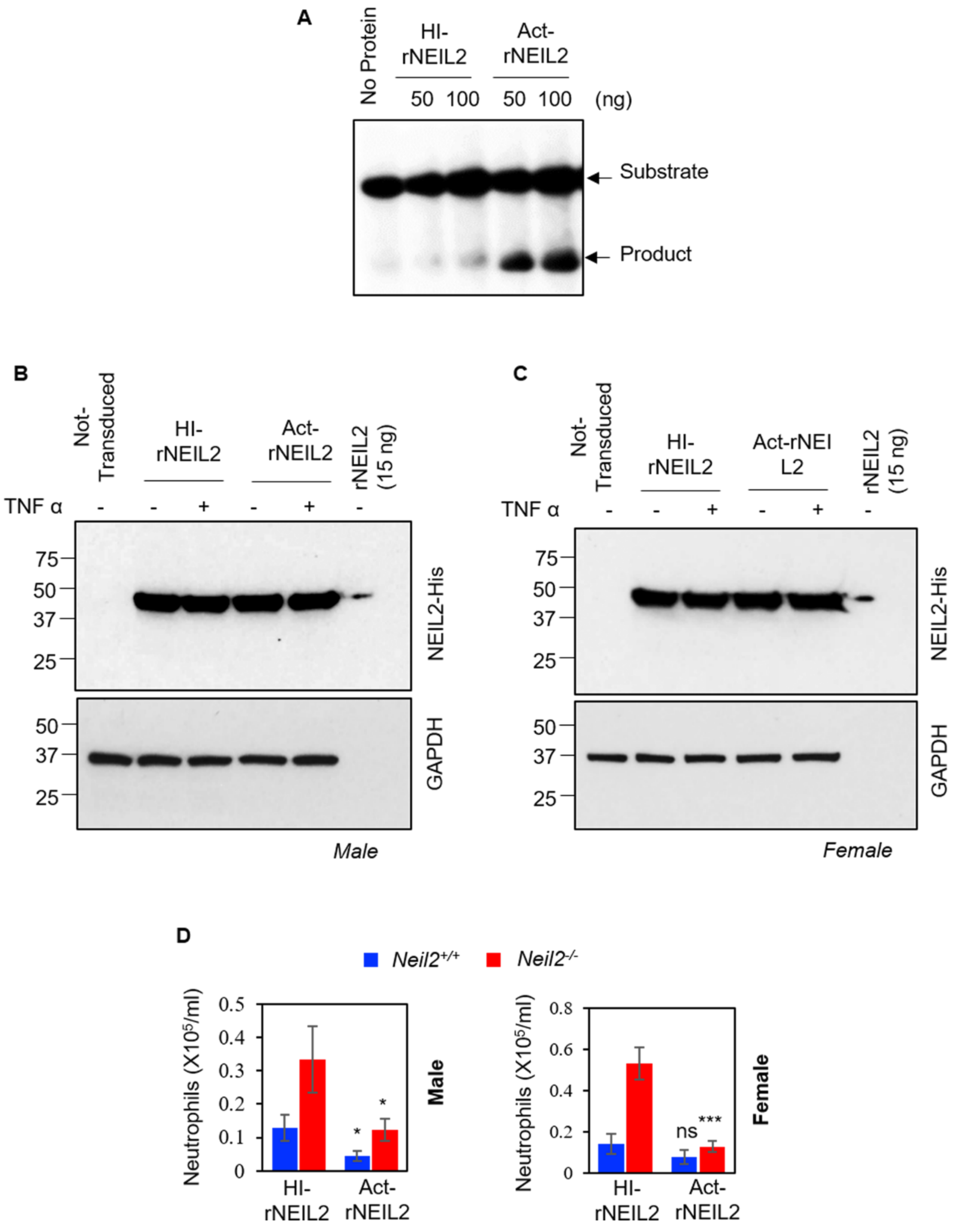

Figure. S5. Intrapulmonary rNEIL2 delivery in *Neil2*$^{+/+}$ or *Neil2*$^{-/-}$ male and female mice.

(**A**) Analysis of glycosylase activity of HI-rNEIL2 or Act-rNEIL2 with ^{32}P-labeled 5-hydroxyuracil DNA-bubble substrate (51-mer). The substrate and the cleaved 25-mer product are indicated by arrows. (**B** and **C**) Immunoblots of whole cell extracts from -/+TNFα treated *Neil2*$^{+/+}$ male (B) or female (C) mice lung,

72 h post-transduction with HI-rNEIL2 or Act-rNEIL2 using anti-His and GAPDH antibodies. Act-rNEIL2 protein (15 ng) was loaded as positive control. (**D**) Neutrophil counts post 16 h mock-challenge in broncho-alveolar lavage fluid of *Neil2*$^{+/+}$/*Neil2*$^{-/-}$ male and female mice lung transduced with HI-rNEIL2 or Act-rNEIL2. Error bars represent ± standard deviation from the mean (n=3). ns= not significant; *=p <0.05; **=p <0.01; ***=p <0.005 vs. HI-rNEIL2 transduced group.

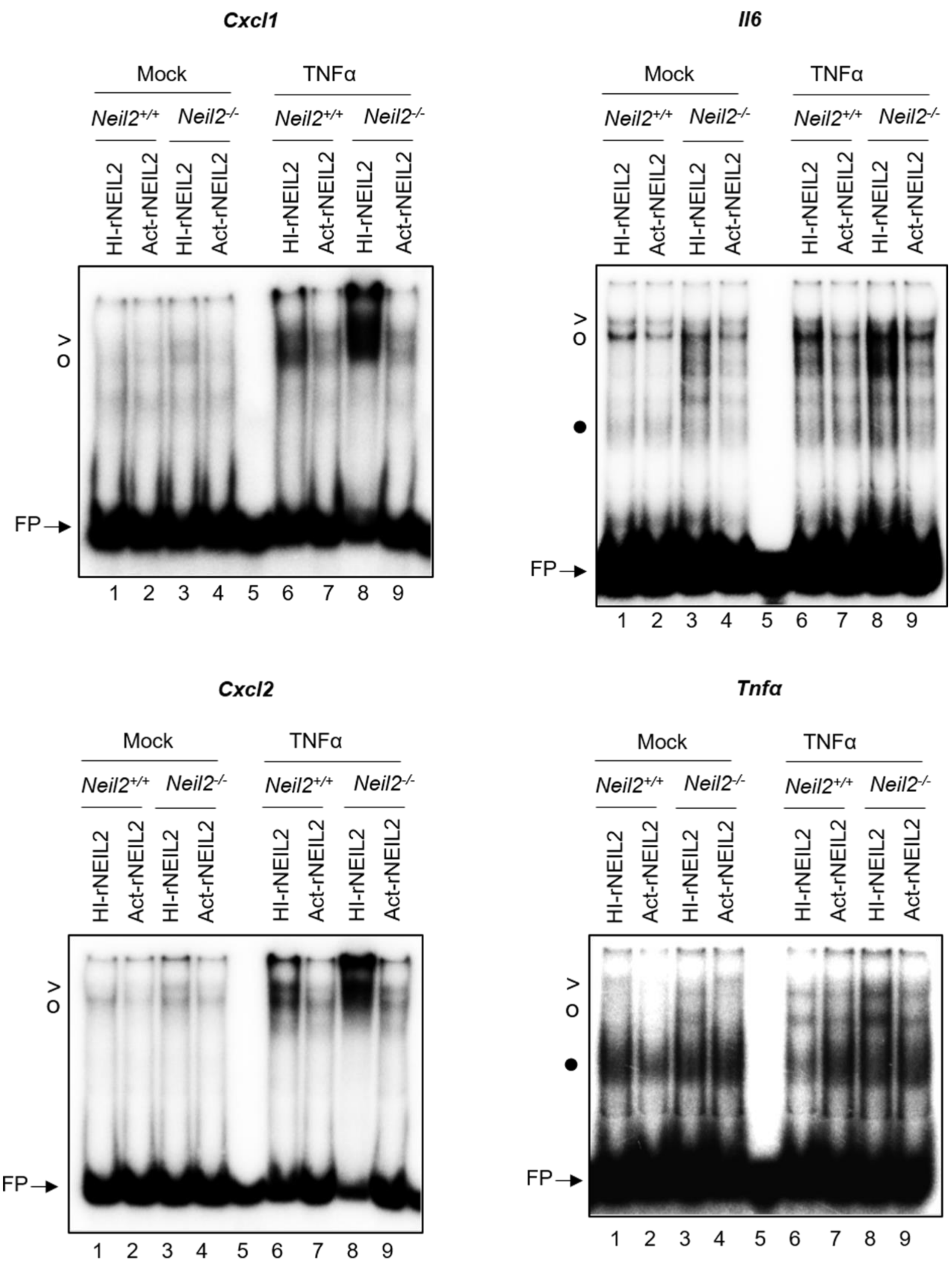

Figure. S6. Electrophoretic mobility-shift assays (EMSAs) with lung nuclear extract from *Neil2*$^{+/+}$ or *Neil2*$^{-/-}$ male mice.

Full size EMSA images for Figure 5E are displayed here. EMSAs of NF-κB binding using nuclear extracts pooled from *Neil2*$^{+/+}$/*Neil2*$^{-/-}$ male mice lung (n=3), transduced with HI-rNEIL2 or Act-rNEIL2 prior to

mock/TNFα-exposure, with radiolabeled probes derived from *Cxcl1, Cxcl2, IL6* and *Tnfα* promoters as indicated on the top; Representatives of three independent gels are shown; > and ^{O} denote RelA-DNA complexes; ● denotes nonspecific complex; FP represents free probe. lane 5: probe only.

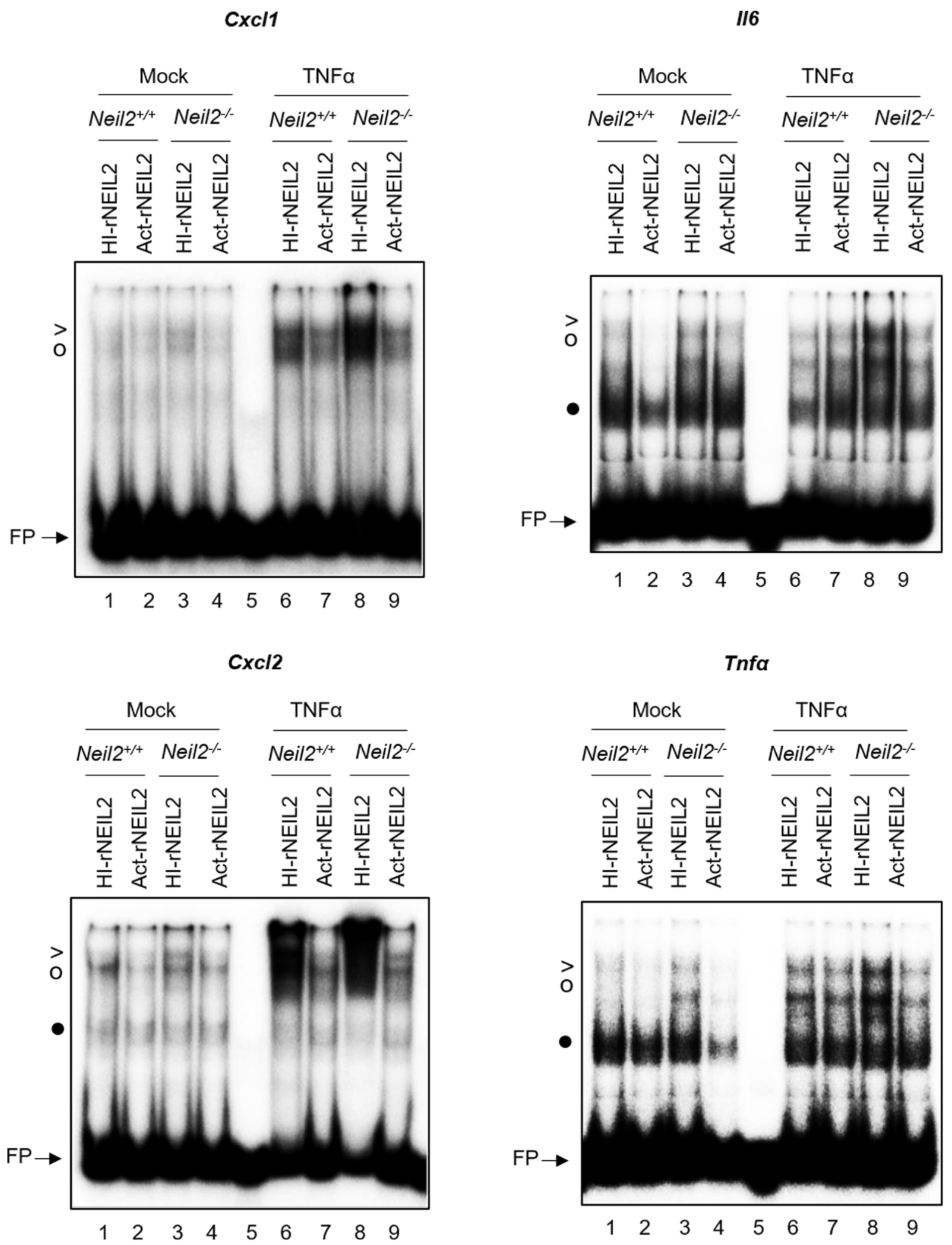

Figure. S7. Electrophoretic mobility-shift assays (EMSAs) with lung nuclear extract from *Neil2*$^{+/+}$ or *Neil2*$^{-/-}$ female mice.

EMSA of NF-κB binding using nuclear extracts pooled from *Neil2*$^{+/+}$/*Neil2*$^{-/-}$ female mice lung (n=3), transduced with HI-rNEIL2 or Act-rNEIL2 prior to mock/TNFα-exposure, with radiolabeled probes

derived from *Cxcl1*, *Cxcl2*, *IL6* and *Tnfα* promoters as indicated on the top; Representatives of three independent gels are shown; > and ^{O} denote RelA-DNA complexes; ● denotes nonspecific complex; FP represents free probe. lane 5: probe only.
